# Epigenomic evolution in diffuse large B-cell lymphomas

**DOI:** 10.1038/ncomms7921

**Published:** 2015-04-20

**Authors:** Heng Pan, Yanwen Jiang, Michela Boi, Fabrizio Tabbò, David Redmond, Kui Nie, Marco Ladetto, Annalisa Chiappella, Leandro Cerchietti, Rita Shaknovich, Ari M. Melnick, Giorgio G. Inghirami, Wayne Tam, Olivier Elemento

**Affiliations:** 1 Institute for Computational Biomedicine, Department of Physiology and Biophysics, Weill Cornell Medical College, 1300 York Avenue, New York, New York 10021, USA; 2 Institute for Precision Medicine, Weill Cornell Medical College, 1300 York Avenue, New York, New York 10021, USA; 3 Hematology/Oncology Division, Department of Medicine, Weill Cornell Medical College, 1300 York Avenue, New York, New York 10021, USA; 4 Department of Pathology and Laboratory Medicine, Weill Cornell Medical College, 1300 York Avenue, New York, NY 10021, USA; 5 Department of Molecular Biotechnology and Health Science and Center for Experimental Research and Medical Studies (CeRMS), University of Torino, Torino 10126, Italy; 6 Divisione di Ematologia, Azienda Ospedaliera Santi Antonio e Biagio e Cesare Arrigo, Alessandria 10126, Italy; 7 Division of Hematology, Azienda Ospedaliero Universitaria Città della Salute e della Scienza di Torino, Turin 10126, Italy

## Abstract

The contribution of epigenomic alterations to tumour progression and relapse is not well characterized. Here we characterize an association between disease progression and DNA methylation in diffuse large B-cell lymphoma (DLBCL). By profiling genome-wide DNA methylation at single-base pair resolution in thirteen DLBCL diagnosis–relapse sample pairs, we show that DLBCL patients exhibit heterogeneous evolution of tumour methylomes during relapse. We identify differentially methylated regulatory elements and determine a relapse-associated methylation signature converging on key pathways such as transforming growth factor-β (TGF-β) receptor activity. We also observe decreased intra-tumour methylation heterogeneity from diagnosis to relapsed tumour samples. Relapse-free patients display lower intra-tumour methylation heterogeneity at diagnosis compared with relapsed patients in an independent validation cohort. Furthermore, intra-tumour methylation heterogeneity is predictive of time to relapse. Therefore, we propose that epigenomic heterogeneity may support or drive the relapse phenotype and can be used to predict DLBCL relapse.

## Abstract (web summary)

The contribution of epigenomic alterations to tumour progression and relapse is not well characterized. Here the authors characterize epigenetic evolution in aggressive B-cell lymphoma and find that epigenomic heterogeneity may not only support and drive the relapse phenotype but also be used to predict lymphoma relapse.

## Graphical abstract

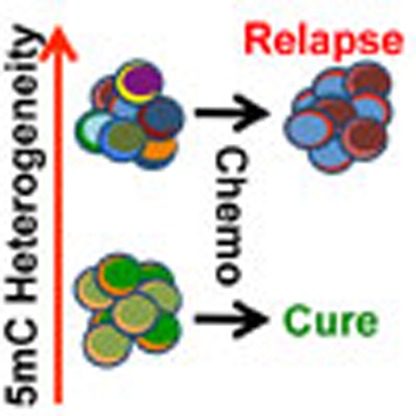

Diffuse large B-cell lymphoma (DLBCL) is the most prevalent B-cell non-Hodgkin lymphoma in adults worldwide^1^,^2^. One-third of patients do not respond to chemotherapy (R-CHOP, rituximab, cyclophosphamide, doxorubicin, vincristine and prednisone) or relapse in 5 years after treatment^3^,^4^. The disease is characterized by heterogeneous genetic, phenotypic and clinical features^5^,^6^, which could only partially explain the failure treatment of some patients. Recent large-scale genomic studies have shown that mutations in the epigenetic machinery and concomitant perturbation of epigenomic patterning are frequent events in B-cell lymphomas^7^. The best-studied epigenetic modification is DNA methylation, which consists of the addition of a methyl group to carbon 5 of the cytosine within the dinucleotide CpG^8^. Hyper- or hypomethylation of gene regulatory regions is associated with gene silencing or expression, respectively. Promoter methylation is generally inversely correlated with gene expression level. DNA methylation-profiling studies indicate that cytosine methylation distribution is perturbed in lymphomas compared to normal B cells^7^. For example, hypermethylation of a CpG-rich region within the first intron of BCL6 was reported to maintain high level of expression of the critical oncogene in part by blocking binding of a negative regulator CTCF of this locus^9^.

While the contribution of DNA methylation to lymphomagenesis has been investigated, the role of DNA methylation in lymphoma progression and relapse is unknown. In childhood acute lymphoblastic leukaemia, a tendency towards hypomethylation was demonstrated in relapsed tumours by whole-methylome analyses^10^,^11^. In DLBCL, inhibition of DNA methylation using azacitidine can overcome chemotherapy resistance both in preclinical models and possibly in patients^12^. These results may implicate DNA methylation in resistance to standard treatment in DLBCL, a feature also observed in relapsed disease. It has been reported that aberration in DNA methylation increases with disease severity in B-cell lymphomas, suggesting again a potential role for DNA methylation in B-cell lymphoma progression^13^. Here we reason that characterizing the DNA methylome at single-base resolution in relapsed DLBCLs would help understand its role in disease progression and its contribution to relapse-associated phenotypes such as chemoresistance. We performed enhanced reduced representation bisulfite sequencing (ERRBS)^14^ to interrogate the methylation levels at three to four million CpGs distributed in a cohort of DLBCL patients who were uniformly treated with standard chemotherapy (R-CHOP) and eventually relapsed. Taking advantage of the single-nucleotide resolution provided by ERRBS^14^, we are able to access intra-tumour methylation heterogeneity (MH) at diagnosis and relapse in DLBCL and correlate it with disease progression.

We characterize the evolution of methylome from diagnosis to relapse in DLBCL along three different axes—overall changes in DNA methylation landscape, differentially methylated regulatory elements and intra-tumour MH. We find that DNA methylation landscape evolves in heterogeneous ways from diagnosis to relapse in our cohort, which is consistent to the complex genetic background of DLBCL. Despite the heterogeneous background of DNA methylation landscape, we determine a methylation signature based on consistently differentially methylated regulatory elements between diagnosis and relapsed pairs. This signature is linked with specific genes and pathways whose role in relapse may be important, for example, transforming growth factor-β (TGF-β) receptor activity pathway and apoptosis. Finally, we observe decreased intra-tumour MH from normal tissues to primary diagnosis tumour samples then to relapsed tumour samples. We also compare and contrast the tumour methylome of the relapsed cohort with another group of patients (*n*=7) who have not relapsed in 5 years after initial treatment. We conclude that non-relapsed patients have lower intra-tumour MH at diagnosis compared with relapsed ones. Importantly, this conclusion has been validated in a second, independent and larger (*n*=59) cohort of DLBCL patients. Our results provide insights into the evolution of the DLBCL epigenome on chemotherapy and how DNA methylation may help to drive the relapse phenotype. Our data also suggest that epigenetic heterogeneity in DLBCLs at diagnosis is predictive of the occurrence of relapse.

## Results

### Heterogeneous DNA methylation changes on DLBCL relapse

**Figure 1:**
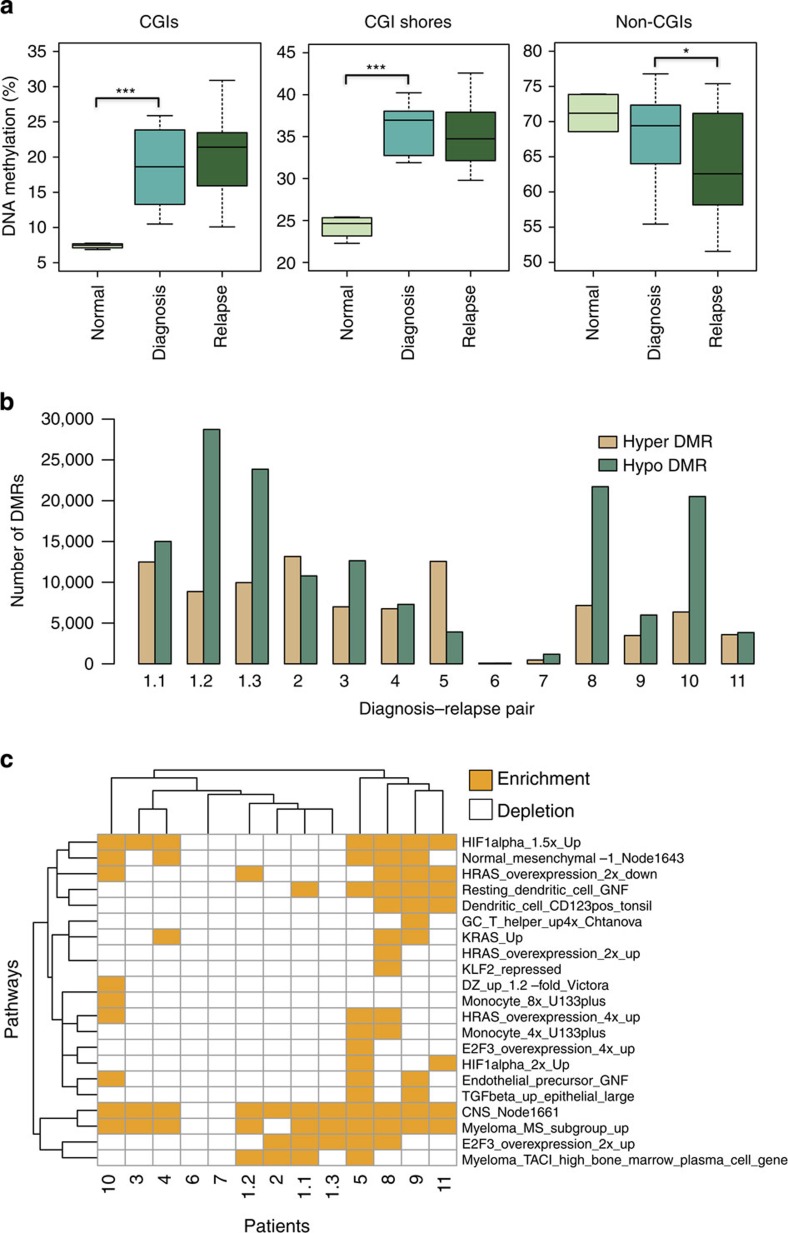
DNA methylation landscape changes in DLBCL patients. (**a**) Percentage of DNA methylation in CGIs, CGI shores and non-CGIs of normal B cells (*n*=4) and diagnosis–relapse DLBCL sample pairs (*n*=13). For each CpG, we collected the number of methylated reads and the number of total reads. The DNA methylation for different genomic regions for each sample was calculated by the percentage of methylated reads out of total reads from all the CpGs inside corresponding regions. ***P<1e−5, *P<0.05 (*t*-test, normal versus diagnosis; paired *t*-test, diagnosis versus relapse). The median, upper and lower quartiles are shown. Whiskers represent upper quartile+1.5 IQR and lower quartile−1.5 IQR. (**b**) Numbers of hypermethylated or hypomethylated DMRs of individual DLBCL patients, between diagnosis and relapse. Patient 1 had 3 sites of relapse and consequently 3 diagnosis–relapse pairs were analysed (the same diagnosis sample was used for all comparisons). Out of 13, 11 pairs show more hypomethylated regions than hypermethylated regions. (**c**) Pathways overexpressed with hypermethylated genes (promoters overlapped with hypermethylation DMRs) of individual patients were illustrated here. Each row represents a single pathway and each column represents a patient pair. The enrichment of the pathways was determined in a patient-by-patient manner (*n*=1, *P*<0.005, randomization-based non-parametric testing). GO analyses were performed with iPAGE^20^. Pathways from the lymphoid gene signature database for Staudt Lab^21^ were used here. The background included around 24,000 genes from Refseq. IQR, interquartile range.

Several studies have shown that the DNA methylation landscape differs between primary DLBCL (at diagnosis) and normal B cells including germinal centre B cells (GCBs) and naïve B cells (NBs)^13^,^15^,^16^,^17^. Here we sought to determine whether DLBCL progression and relapse are associated with DNA methylation landscape changes. To characterize the DNA methylome, we performed ERRBS^14^ on 13 pairs of DLBCL diagnosis tumours (untreated) and their matched relapsed (after treatment) samples from 11 patients. Time to relapse varied between 0.5 and 13 years (Cohort 1; Supplementary Data 1). In one patient, we obtained tissues from three different sites of relapse and performed ERRBS on all three relapsed samples. Approximately half of our samples were of the GCB subtype at diagnosis as assessed by immunohistochemistry (Cohort 1; Supplementary Data 1). In all but one case, the relapsed tumour was of the same subtype as the diagnosis tumour. In addition to tumours, we profiled methylomes of tonsilar B-cell subsets (GCBs and NBs) from two healthy individuals. ERRBS provided more than 10 × sequencing coverage (centred around 50 ×) on three to four million CpG sites genome wide for each sample (Supplementary Data 2). These data sets were used to calculate the DNA methylation levels for all CpG islands (CGIs) as well as outside of CGIs. Hierarchical clustering of CGI methylation levels in all samples revealed that diagnosis and relapse samples from the same patient always cluster together but nonetheless showed that significant methylation differences exist between sample pairs (Supplementary Fig. 1). When analysing the methylation levels from all patients, we observed increased DNA methylation levels at CGIs (*P*=6.9e−5, *t*-test; *P*=1.5e−3, Wilcoxon test) and CGI shores (defined as 1 kb flank regions of known CGIs on both sides; *P*=5.1e−7, *t*-test; *P*=1.5e−3, Wilcoxon test) in DLBCLs compared with normal B cells (NBs and GCBs were merged into a single group due to limited sample numbers; Fig. 1a). However, we did not observe significant methylation level changes outside of CGIs (>10 kb away from known CGIs; *P*=0.22, *t*-test; *P*=0.66, Wilcoxon test; Fig. 1a). We chose 10 kb as our cutoff to define regions unambiguously outside of CGIs and GGI shores. These results are in agreement with previous studies in DLBCL^13^,^16^,^18^. When we interrogated the DNA methylation changes between diagnosis and relapsed tumour samples in each pair, we only observed decreased DNA methylation levels outside of CGIs (*P*=0.04, paired *t*-test; *P*=0.07, paired Wilcoxon test) from diagnosis to relapse but no significant DNA methylation changes at CGIs (*P*=0.47, paired *t*-test; *P*=0.59, paired Wilcoxon test) and CGI shores (*P*=0.29, paired *t*-test; *P*=0.08, paired Wilcoxon test; Fig. 1a). These results differ from those observed in treatment-resistant prostate cancer^19^, where DNA methylation increased substantially at CGIs on tumour progression. They however are concordant with those observed in acute lymphoblastic leukaemia^10^,^11^.

The analyses described so far were performed using the average methylation level across all CGIs, CGI shores and non-CGIs. When we interrogated CpG individually, we found that the methylation status of many CpGs changed between diagnosis and relapse differentially. Specifically, we observed between 39,808 and 1,035,960 differentially methylated CpGs (DMCs) in each sample pair (false discovery rate=20%, Fisher Exact test; Supplementary Data 3). In addition, we identified between 78 and 13,162 hypermethylated differentially methylated regions (DMRs) in each case (Fig. 1b and Supplementary Data 3; see Methods for details on how DMRs are determined). The number of hypomethylated DMRs varied between 92 and 28,744 (Fig. 1b and Supplementary Data 3). We selected top 1,000 DMCs (based on adjusted Fisher *P* values) and DMRs (based on methylation difference) from patient 1.1 as representative examples listed in Supplementary Data 4 and 5. Further analyses demonstrated that the tendency toward hypo- or hypermethylation is not affected by the distribution of methylation sites in the genome or by the bias of ERRBS assay towards CG-rich regions (Supplementary Note 1; Supplementary Fig. 2).

Consistent with the global trend towards hypomethylation at relapse, 11 pairs showed more hypomethylated DMRs at relapse, while the other two showed more hypermethylated DMRs, which suggests once again hypomethylation as a potential hallmark of DLBCL relapse (Fig. 1b). Despite this trend, the number of DMRs within each pair varied broadly between cases (Fig. 1b). In patient 1 for whom we had three sites of relapse, we found that two of the sites have similar methylation profiles suggesting recent divergence, while the other one had significantly different methylation landscape (Fig. 1b and Supplementary Fig. 1). We applied pathway analysis using the information-theoretic iPAGE^20^ approach to the genes associated with hypermethylated and hypomethylated DMRs at relapse (these genes are defined as genes with promoters that overlapped with DMRs) in each patient separately (see Methods). We identified a small number of pathways that are over-represented among hypermethylated DMR-associated genes in more than two patients, such as CNS_Node1661 (ref. 21; central nervous system cell differentiation pathways; Fig. 1c). However, most differentially methylated pathways in relapse are specific to one or two individuals such as E2F3_overxpression_4x_up^21^ (genes upregulated by E2F3 by fourfold; Fig. 1c). Altogether these results indicate distinct evolutionary directions of the DNA methylome in DLBCL patients, with each patient evolving along its own trajectory.

### Preferential methylation changes at regulatory elements

**Figure 2:**
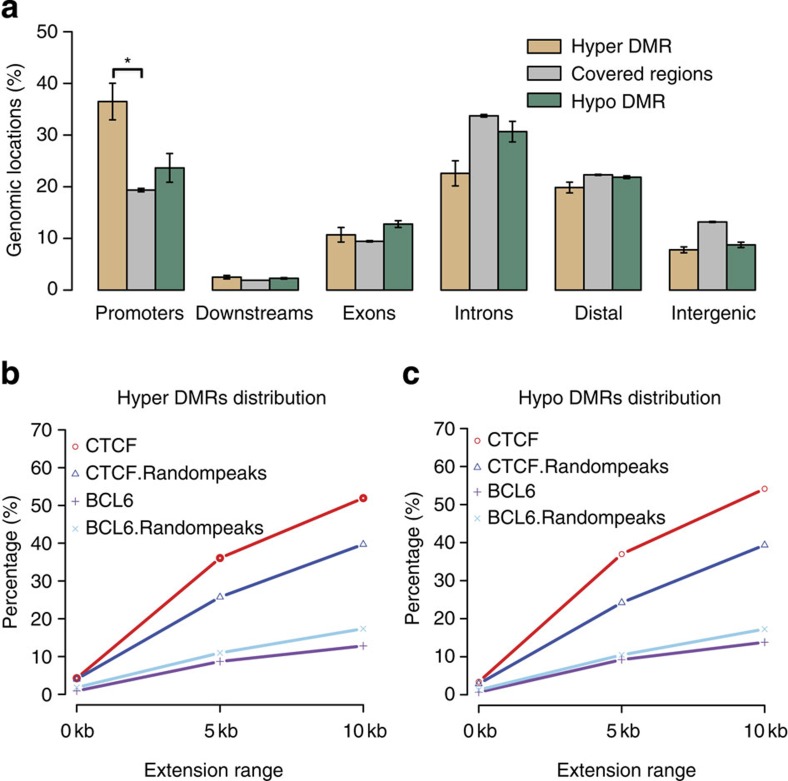
Consistently differentially methylated regulatory elements between diagnosis and relapsed patients. (**a**) Percentage of hypermethylated regions, ERRBS-covered regions and hypomethylated regions within indicated genomic locations. *P<2.2e−16 (binomial test). Standard errors are shown as error bars. (**b**) Percentage of hypermethylated DMRs occurring within CTCF peaks, CTCF random peaks, BCL6 peaks and BCL6 random peaks. (**c**) Percentage of hypomethylated DMRs occurring within CTCF peaks, CTCF random peaks, BCL6 peaks and BCL6 random peaks. In **b**,**c**, random peaks were generated randomly with the same genomic distribution as the true binding sites^22^.

We sought to determine where in the genome DNA methylation changes tend to occur between diagnostic and relapsed samples. Across all patients, we found that hypermethylated DMRs at relapse were mostly enriched at promoters (defined as ±2 kb windows centred on RefSeq transcription start sites) compared with the distribution of ERRBS-covered regions (36 versus 19%, *P*=2.2e−16, binomial test; *P*=4e−4, *t*-test; Fig. 2a). These regions were defined as containing at least five covered CpGs minimum, separated by <250 bp between contiguous CpGs, which is similar to the definition of DMRs without methylation ratio cutoff and DMCs requirement. The distribution of hypomethylated DMRs was overall similar to the distribution of ERRBS-covered regions (Fig. 2a). Next we examined the methylation changes at or nearby CTCF-binding sites between diagnostic and relapsed samples. For this analysis, we used CTCF sites determined by chromatin immunoprecipitation (ChIP)-seq in the OCI-Ly1 DLBCL cell line. We observed that 52% of hypermethylated DMRs and 54% of hypomethylated DMRs were located in the neighbourhood (≤10 kb) of CTCF-binding sites (Fig. 2b,c). As a control, we generated randomly located CTCF-binding sites with the same genomic distribution as the true binding sites^22^ and found significantly fewer relapse-associated hypermethylated (40%, *P*=2.2e−16, Fisher Exact test) and hypomethylated DMRs (39%, *P*=2.2e−16, Fisher Exact test) in close proximity to these random sites. These results support a link between CTCF binding and DNA methylation changes at relapse. As another control, we performed the same analysis on BCL6-binding sites in OCI-Ly1 obtained from a previous study^23^. BCL6 has not been reported to be associated with DNA methylation status and accordingly we did not observe any significant association between DMRs and BCL6 binding. Altogether these results confirm a potential mechanistic connection between DNA methylation evolution in DLBCL and specific regulatory elements.

### Methylation signature at relapse involves key pathways

Despite the high patient-to-patient evolutionary heterogeneity in DNA methylation, we hypothesize that there might be a small but common methylation signature of DLBCL relapse in which the evolutionary relapse methylome converges at specific genes. To determine such a methylation signature, we specifically focused on three kinds of genomic locations:promoters, enhancers and CTCF-binding sites. We selected these regulatory elements because methylation changes occur within them as shown above and methylation at these elements is known to impact gene regulation^24^,^25^,^26^. To determine a methylation signature, we performed supervised analysis of the DNA methylation levels of regulatory elements. We labelled samples as diagnosis and relapse and sought to call differentially methylated elements between these two groups using a paired statistical approach. In addition to promoters, we used enhancers derived from the OCI-Ly1 cell line using ChIP-seq and defined as regions positive for H3K4me2 and H3K27Ac but not H3K4me3 (ref. 23; since we could not perform histone modification ChIP-seq due to limited availability of patient-derived tumour tissues) We also used CTCF sites determined by ChIP-seq in the OCI-Ly1 DLBCL cell (see Methods). Altogether we identified 107 differentially methylated promoters, 22 enhancers and 118 CTCF-binding sites between diagnosis and relapse (>10% DNA methylation changes and *P*<0.05, paired *t*-test, two-sided; Supplementary Fig. 3, Supplementary Fig. 4a–c and Supplementary Data 6). Next we associated differentially methylated regulatory elements with genes based on genomic proximity (see Methods). In total, we determined 44 hypermethylation related genes and 490 hypomethylation related ones (Supplementary Note 1). Many genes in our signature are potentially related to tumour progression. For example, *SMAD6* (near hypomethylated CTCF-binding site) is one of the regulators of TGF-β superfamily pathway, which plays a key role in lymphoma biology^27^.

**Figure 3:**
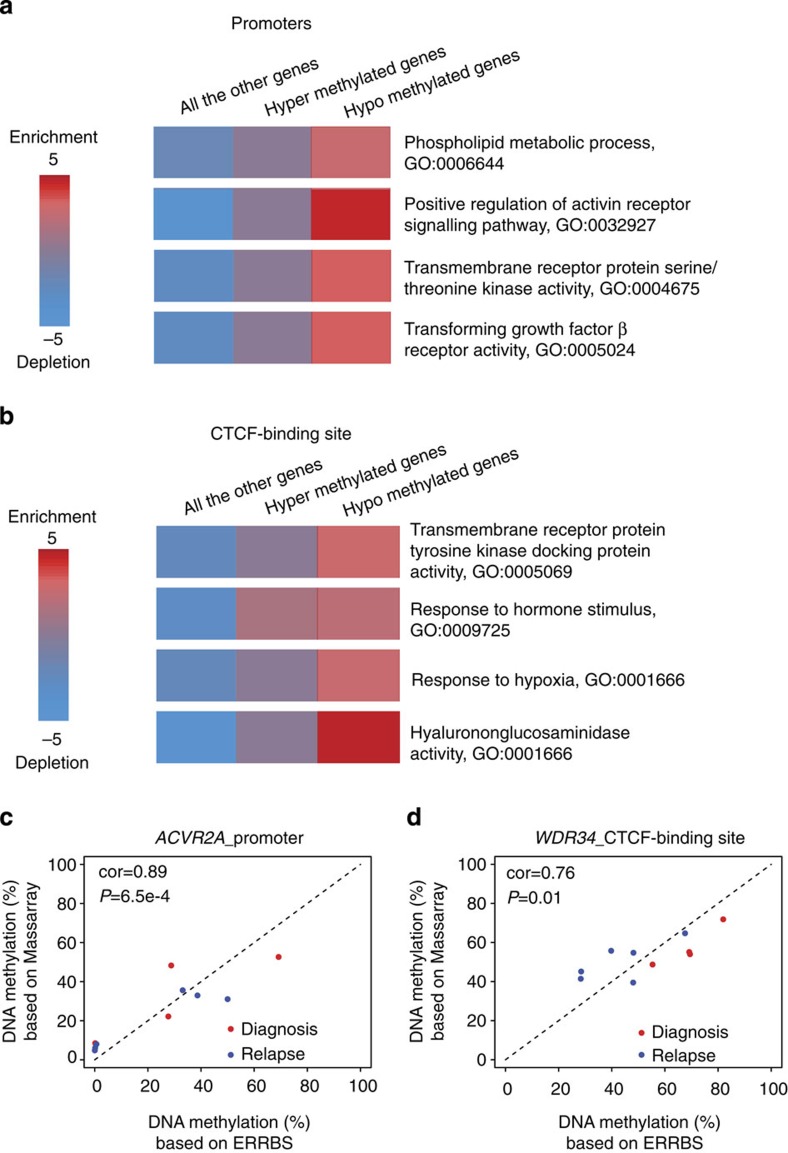
Methylation signature at relapse involves key genes and pathways. (**a**) Pathways over-represented within consistently differentially methylated genes (based on promoters) across all the patients. *P* values were 0.0030, 0.0001, 0.0005 and 0.0009 from top to bottom (hypergeometric tests). (**b**) Pathways over-represented within genes in the neighbourhood of hypomethylation or hypermethylation CTCF peaks (≤10 kb). *P* values were 0.0036, 0.0024, 0.0029 and 0.0000 from top to bottom (hypergeometric tests). In **a**,**b**, GO analyses were performed with iPAGE^20^. Known pathways in the GO^46^ were used here. The background included around 24,000 genes from Refseq gene annotation. The red colour indicates (in log_10_) the over-represented *P* values and the blue shows under-representation. (**c**) Correlations of average methylation level between ERRBS and MassArray in *ACVR2A* promoter. (**d**) Correlations of average methylation level between ERRBS and MassArray in CTCF-binding site near *WDR34*. In **c**,**d**, red and blue dots represent diagnosis and relapsed samples, respectively. *P* values were derived from correlation test (cor.test() function in *R*).

To confirm the functionality of our methylation signature genes, we first performed pathway analysis with iPAGE^20^ on the corresponding genes and identified several pathways as over-represented among hyper- or hypomethylated genes, notably including anti-apoptosis and tumour necrosis factor activity (Fig. 3a,b and Supplementary Fig. 5). Activin receptor activity was for example over-represented in hypomethylated genes with two genes *ACVR2A* and *ACVR2B* being frequently hypomethylated (Supplementary Fig. 4a). *ACVR2A* and *ACVR2B* have important roles in apoptosis, immune response, cell proliferation and differentiation and may therefore play a role in lymphoma relapse^27^,^28^,^29^. Genes associated with TGF-β receptor activity were over-represented among hypomethylated genes (Fig. 3a). It has been previously reported that escaping from TGF-β-mediated growth inhibition is critical to lymphoma relapse^27^. Our data suggest that methylation aberrations of genes in the TGF-β receptor activity pathway might be involved in lymphoma relapse, thus confirming another recent report linking methylation of TGF-β-associated genes with chemoresistance^12^. In one patient where we had sufficient tissue, we performed RNA-seq on diagnostic and relapsed biopsies and found in that patient hypomethylation related genes (genes whose promoter is located nearby a DMR, within 5 kb) were correlated significantly with higher gene expression level (*P*=2.2e−16, *t*-test; Supplementary Fig. 6). Altogether these results suggest that our methylation signature is associated with DNA methylation changes and may help mediate the relapse phenotypes by contributing to the enhanced expression of key genes.

To validate the robustness of our methylation signature, we used an orthogonal approach (MassArray) to validate the methylation levels of our ERRBS identified elements. We performed MassArray on nine randomly selected regions (three promoters, three enhancers and three CTCF-binding sites) from 10 samples to validate the average methylation levels of these regions in both diagnostic and relapsed samples. We observed that seven out of nine regions displayed significant correlations of average methylation level between ERRBS and MassArray (*P*<0.05), including *ACVR2A* promoter and *WDR34* CTCF-binding site (Fig. 3c,d and Supplementary Data 7).

### Intra-tumour MH decreases at relapse

**Figure 4:**
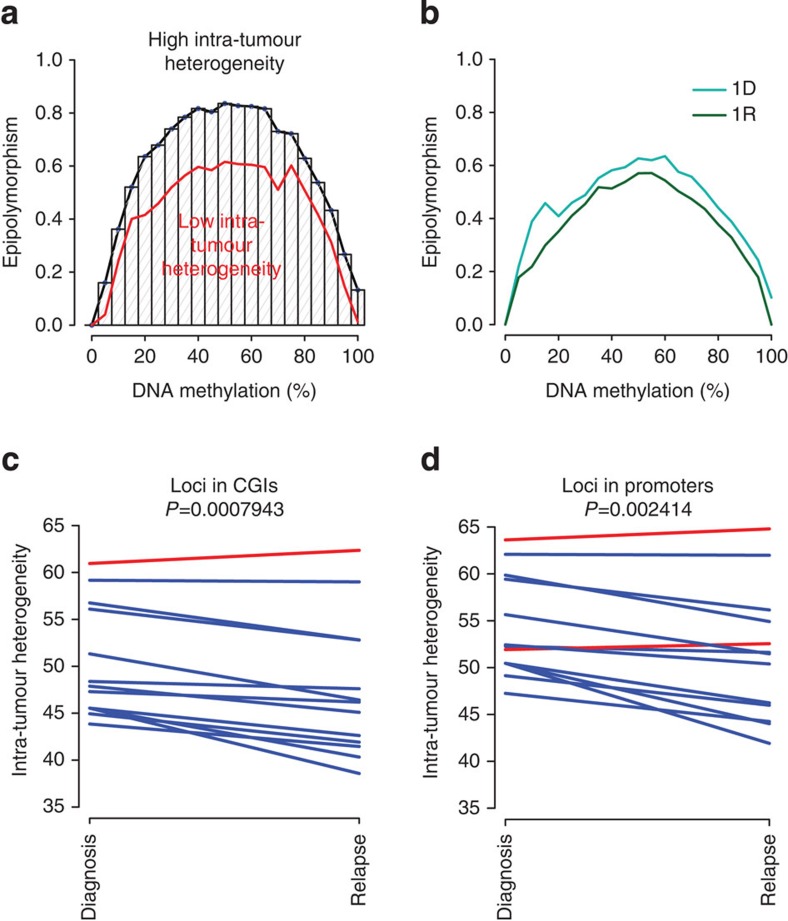
Intra-tumour MH decreases from diagnosis to relapse in DLBCL patients. (**a**) Epipolymorphism levels are dependent on DNA methylation levels. All the loci were divided into different groups based on their methylation level and median epipolymorphism of each group is calculated. Genome-wide intra-tumour MH was quantified by area under the median line. (**b**) Median epipolymorphism lines for diagnosis and relapse tumors from patient 1.1 in our cohort. Intra-tumor MH visibly decreased with tumour evolution. (**c**) Relapsed samples displayed significant lower intra-tumour MH. Out of 13, 12 pairs displayed lower intra-tumour MH. All the loci were located in CGIs. (**d**) Relapsed samples displayed significant lower intra-tumour MH. All the loci located in gene promoter. Out of 13, 11 pairs displayed lower intra-tumour MH. In **c**,**d**, intra-tumour MH was measured by area under median epipolymorphism line in sample-by-sample manner. *P* values were obtained from paired *t*-test of intra-tumour MH between diagnosis and relapsed samples.

We next investigated the association between intra-tumour MH and tumour evolution. Bisulfite sequencing-based DNA methylation profiling provides a unique opportunity to quantify intra-tumour DNA MH since each sequenced read is derived from one individual tumour cell (Supplementary Fig. 7). As shown in Supplementary Fig. 7, loci with identical DNA methylation level can have varying levels of intra-tumour MH. To assess the intra-tumour MH of specific genomic region, we used the concept of epipolymorphism as recently introduced in Landan *et al.*^30^. In brief, the epipolymorphism of a given locus was defined as the probability that two epialleles found at that locus and randomly sampled from the cell population differ from each other. To calculate a genome-wide measure of MH, we binned epipolymorphism values for all loci genome-wide according to methylation level. The median epipolymorphism level across the range of methylation levels reflects epigenetic heterogeneity genome wide (Fig. 4a and Methods) and enables comparisons between diagnostic and relapsed samples. For example, the intra-tumour MH measured by epipolymorphism decreased at relapse for patient 1.1 (Fig. 4b; Supplementary Fig. 8). Overall, we observed that intra-tumour MH decreased significantly from diagnosis to relapse across all sample pairs except Patient 6 (*P*=8e−4, paired *t*-test; *P*=0.003, paired Wilcoxon test; Fig. 4c). We found that epipolymorphism decreased in most methylation bins in all sample pairs except patient 6 (Supplementary Data 8). This result is consistent with clonal selection of a subset of lymphoma cells on chemotherapy treatment leading to the relapsed tumour. This observation is robust when we interrogated other groups of loci in promoters (with different selection standards) in the intra-tumour MH. When we chose loci located within promoter regions, we found that 11 out of 13 pairs displayed significant lower intra-tumour heterogeneity (*P*=0.002, paired *t*-test; *P*=0.003, Wilcoxon test; Fig. 4d). Of note, we also observed lower intra-tumour MH in diagnostic samples compared with normal tissues both in CGIs (*P*=0.0002, *t*-test; *P*=0.003, Wilcoxon test) and in promoters (*P*=0.0004, *t*-test; *P*=0.006, Wilcoxon test; Supplementary Fig. 9a,b). These results are consistent with normal B cells consisting of a population of diverse, non-clonal B cells with distinct methylation patterns and DLBCL tumours arising from individual B-cell clones.

**Figure 5:**
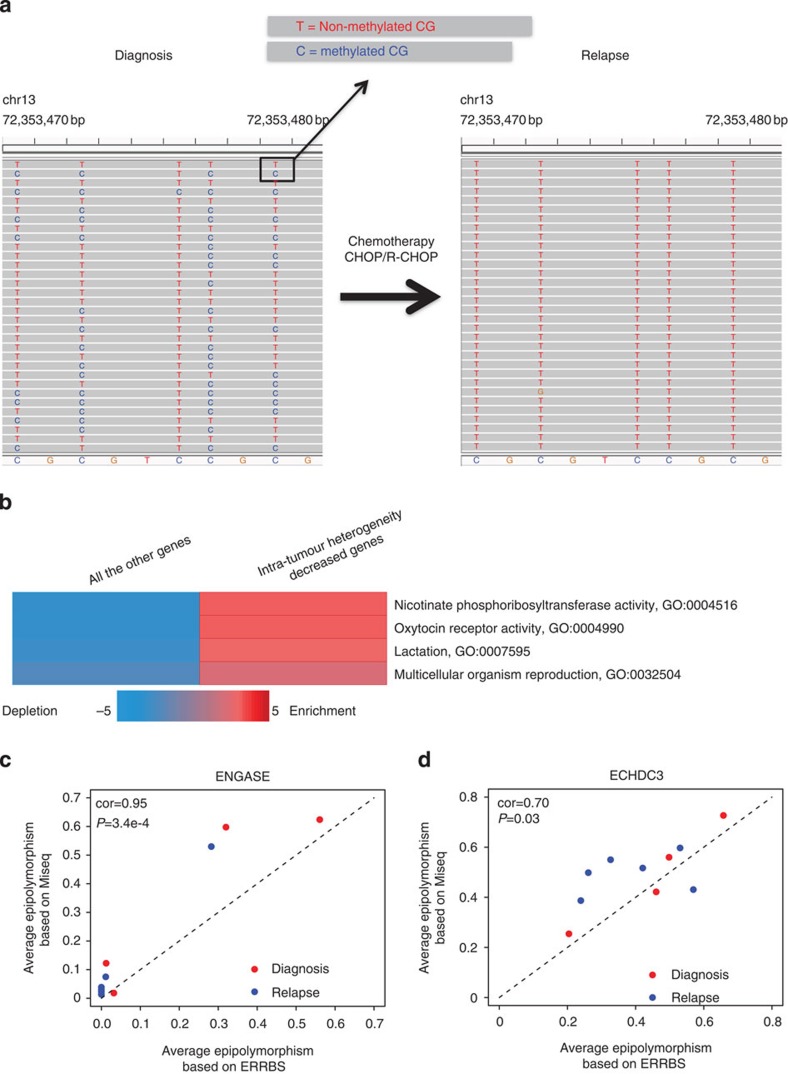
Convergence of methylation patterns at relapse involves key genes and pathways. (**a**) A locus displayed decreased intra-tumour MH from diagnosis to relapse. T and C at CpG site indicate unmethylated and methylated CpG separately. The tumour cell population displayed diverse DNA methylation patterns at diagnosis and the diversity is complete loss at relapse. (**b**) Pathways over-represented with decreased intra-tumour MH genes. *P* values were 0.0005, 0.0005, 0.0015 and 0.0048 from top to bottom (hypergeometric tests). GO analyses were performed with iPAGE^20^. Known pathways in the GO^46^ were used here. The background included around 24,000 genes from Refseq gene annotation. The red colour indicates (in log_10_) the over-represented *P* values and the blue shows under-representation. (**c**) Correlations between the average intra-tumour MH derived from ERRBS or Bisulfite-PCR-MiSeq in *ENGASE* promoters. (**d**) Correlations between the average intra-tumour MH derived from ERRBS or Bisulfite-PCR-MiSeq in *ECHDC3* promoters. In **c**,**d**, MiSeq based intra-tumour MH was calculated using the same analytical approach as the one used for ERRBS. Red and blue dots represent diagnosis and relapsed samples, respectively. *P* values were derived from correlation test (cor.test() function in *R*).

A systematic comparison of intra-tumour MH dynamics at promoters between diagnostic and relapsed samples identified 14 gene promoters that displayed pronounced lower intra-tumour MH from diagnosis to relapse (>5% epipolymorphism decrease and *P*<0.05, paired *t*-test), including *ADCY6*, *C9orf142*, *ECHDC3*, *ENGASE*, *HSPA4L*, *ISL2*, *KCNH3*, *LHX4*, *NAPRT1*, *OXTR*, *PACSIN1*, *PPP1R3G*, *SPIRE2* and *TMEM130.* Using one locus (chr11: 72,353,470–72,353,478) as an example (Fig. 5a), we showed that tumour populations displayed diversified DNA methylation patterns at diagnosis but this diversity was complete lost at relapse. When applying iPAGE^20^ to 14 intra-tumour MH decreased genes, several enriched pathways were identified (Fig. 5b). We postulate that at least some of these promoters are under selection for specific methylation alleles. Generally, more promoters displayed lower intra-tumour MH from diagnosis to relapse than higher heterogeneity (48% versus 40%). It has recently been reported that locally disordered methylation was linked to low-level gene expression in chronic lymphocytic leukaemia^31^. Therefore, decreased MH at relapse may also correlate with gene expression changes.

To validate the robustness of our approach for quantifying intra-tumour MH, we PCR-amplified bisulfite-converted DNA at specific loci and sequenced the PCR product using Illumina MiSeq (PE 2 × 150 bp). We calculated intra-tumour MH using the same analytical approach as the one used for ERRBS. Out of five regions with enough CpGs to compare, we found that four regions had significant correlations between the median intra-tumour MH derived from either ERRBS or Bisulfite-PCR-MiSeq (*P*<0.05; Supplementary Data 9). We used *ENGASE* and *ECHDC3* promoters as examples to illustrate the correlation patterns (Fig. 5c,d).

### Intra-tumour MH predicts relapse

**Figure 6:**
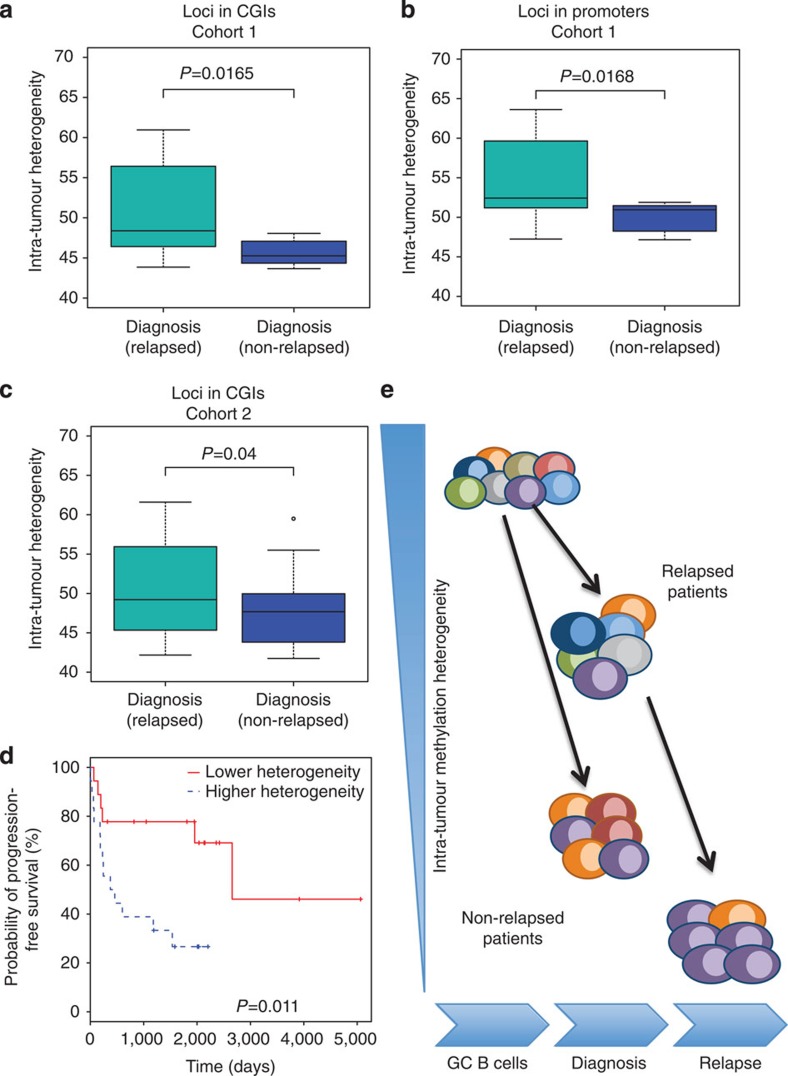
Intra-tumour MH at diagnosis is predictive of relapse occurrence. (**a**) Non-relapsed patients (*n*=7) had lower intra-tumour MH compared with relapsed ones (*n*=11; Cohort 1). All the loci analysed were located in CGIs. (**b**) Non-relapsed patients (*n*=7) had lower intra-tumour MH compared with relapsed ones (*n*=11; Cohort 1). All the loci located in promoters. (**c**) Patients who had not relapsed in 5 years after diagnosis (*n*=19) had lower intra-tumour MH compared with relapsed one (*n*=29; Cohort 2). All the loci analysed here were located in CGIs. In **a**–**c**, *P* values were obtained using *t*-test. The median, upper and lower quartiles are shown. Whiskers represent upper quartile+1.5 IQR and lower quartile−1.5 IQR. (**d**) Kaplan–Meier plot comparing the progression-free survival between DLBCL patients with higher (30%, *n*=18) versus lower (30%, *n*=18) intra-tumour MH (Cohort 2). *P* value was obtained using log-rank test. (**e**) Schematic of our epigenetic evolution model. Different colours represent different cell subgroups with different DNA methylation patterns. Intra-tumour MH decreased with tumour evolution. Non-relapse patients displayed lower intra-tumour MH compared with relapse ones.

We then wondered whether epigenetic heterogeneity could potentially support the Darwinian process in DLBCL tumours. One hypothesis is that tumour cell populations with a large variety of epialleles are more likely to progress and give rise to relapsed tumours than tumours with lower MH. To test this hypothesis, we investigated whether intra-tumour MH at diagnosis would predict whether a DLBCL patient would relapse. To this end, we performed ERRBS on primary tumours from seven DLBCL patients who did not relapse in 5 years after treatment (Cohort 1; Supplementary Data 1). We found that non-relapsed patients displayed significant lower intra-tumour heterogeneity at diagnosis compared with relapsed patients (Cohort 1; loci in CGIs; *P*=0.0165, *t*-test; *P*=0.035, Wilcoxon test; Fig. 6a). We observed similar intra-tumour MH differences when we interrogated promoter loci (Cohort 1; *P*=0.0168, *t*-test; *P*=0.027, Wilcox test; Fig. 6b). When we compared the two DLBCL subtypes (GCB and non-GCB), we found no significant difference in intra-tumour MH between them at CGIs (*P*=0.8, *t*-test; *P*=0.7, Wilcoxon test) or promoters (*P*=0.7, *t*-test; *P*=1, Wilcoxon test). In parallel, we performed VDJ-sequencing for all tumours^32^ and used the clonal frequencies of VDJ somatic hypermutation patterns associated with the main V, D, J rearrangement in each tumour to quantify clonal heterogeneity. In brief, clonal heterogeneity was the empirical entropy calculated by the frequencies of VDJ somatic hypermutation patterns in the tumour population in each patient. In contrast with intra-tumour heterogeneity, we found no significant difference in intra-tumour clonal heterogeneity at diagnosis measured by VDJ-seq between patients who relapsed and patients who did not (*P*=0.29, *t*-test; *P*=0.38, Wilcoxon test; Supplementary Fig. 10). Accordingly, we found no significant correlation between intra-tumour MH and intra-tumour clonal heterogeneity (Pearson cor=−0.2; *P*=0.56). Altogether these results suggest that intra-tumour clonal MH (and not clonal genetic heterogeneity) at diagnosis may be a potential predictor of DLBCL relapse.

To validate our conclusions, we performed ERRBS on a completely independent larger cohort (*n*=59) with extensive clinical annotations (Cohort 2; Supplementary Data 10). As before, ERRBS provided more than 10 × sequencing coverage (50 × on average) on two to three million CpG sites genome-wide for each sample (Supplementary Data 2). In this independent cohort, non-relapsed patients (*n*=19), identified as patients who did not relapse in 5 years after initial diagnosis, displayed significant lower CGIs intra-tumour MH compared with relapsed patients (*n*=29; *P*=0.04, *t*-test; *P*=0.03, Wilcoxon test Fig. 6c). These results independently confirm the data obtained on the first cohort. We then examined progression-free survival after treatment in the 59 patients. Using survival analysis, we found that patients with lower intra-tumour MH (lower 30% of all the patients, *n*=18) were associated with longer progression time (log-rank *P*=0.011; Fig. 6d) compared with high intra-tumour MH group (higher 30% of all the patients, *n*=18). Our results further support the hypothesis that epigenetic heterogeneity within a population of tumour cells at diagnosis might indeed potentiate the Darwinian evolutionary process that leads to relapse (Fig. 6e). Moreover, they further support intra-tumour MH as predictor of relapse occurrence in DLBCL patients.

## Discussion

Many types of genetic lesion, including chromosomal translocations, aberrant somatic hypermutation, point mutations and a variety of copy-number aberrations^33^,^34^, have been identified in DLBCL. Our own work indicates that there is extensive genomic and clonal evolution in DLBCL between diagnosis and relapse^32^. However, genetic lesions do not fully explain the molecular mechanisms underlying tumorigenesis and relapse, and it is therefore reasonable to postulate that epigenetic programming might also contribute to the aggressive and chemoresistant phenotype of relapsed tumours. It is indeed clear that aberrant epigenetic regulation of gene expression is a hallmark of B-cell lymphoma and other types of cancer. In this study, we reason that the examination of DNA methylation profiles could help understand some of the biological and clinical properties of B-cell lymphoma relapse. Accordingly, by analysing the DNA methylation status of 3–4 M CpGs in each sample, we observed highly heterogeneous epigenomic evolutionary trajectories among patients. Nonetheless, we successfully identified a small subset of consistently differentially methylated regulatory elements between diagnosis and relapse and determined a methylation signature based on these elements. While some associated pathways, for example, TGF-β receptor activity pathway have been associated with relapse and chemoresistance by others^12^,^27^, other genes in this signature will require further investigation to determine their exact role in relapse-associated phenotypes such as chemoresistance. Our study also revealed a significant number of distal enhancers that are hypomethylated in relapse and associated with genes involved in pathways such as anti-apoptosis. Enhancer hypomethylation may associate with upregulation of genes involved in a variety of cancer-related pathways^24^. Of note, both promoter and enhancer differential methylation suggest that regulatory sequence activity may evolve in DLBCL as a result of epigenomic changes. This in turn suggests that tumour evolution and relapse may involve rewiring of regulatory networks mediated by epigenomic changes. Also, to our knowledge, the drugs in R-CHOP have not been linked to direct effect on DNA methylation or other epigenetic marks, which confirm the conclusion that the methylome evolution is pathogenetic and relapse related. Such evolution—if confirmed and eventually found to be functional and non-neutral—would parallel the evolution of species, which colleagues in the evo–devo field often ascribe to regulatory network rewiring instead of mutational changes in transcriptional regulators themselves^35^. The limited availability of biopsy tissue precluded the systematic analysis of gene expression changes in the same tumours profiled by DNA methylation. We only performed RNA-seq in one patient and found that in that patient hypomethylation related genes correlated with higher gene expression level (Supplementary Fig. 6). While little can be generalized from only one sample, these results support the overall functionality of the relapse methylation signature identified in this study.

Although our conclusions need to be interpreted with some cautions as based on a relatively small number of samples, many genes in our methylation signature are reported to be associated with tumour progression in lymphomas, including a few robust observations that clearly emerged from published works. In a recent short hairpin RNA screen in DLBCL cell lines, knocking down *ACVR2A* expression in the ABC-DLBCL LY3 cell line^36^ resulted in a two-fold drop in cell viability in at least one hairpin. Another hypomethylated gene in relapse, *TLR3* (member of the Toll-like receptor of pattern recognition receptors of the innate immune system), was associated with a 16-fold decrease in viability on knockdown in the ABC-DLBCL LY10 cell line^36^, in at least one hairpin. One of the anti-apoptotic genes located near a hypomethylated enhancer at relapse was *BCL2*, a prognostic marker for the ABC-DLBCL subtype^37^. *E2F4* has been reported to be downregulated in Burkitt lymphoma and upregulated in DLBCL^38^. Hypoxia-inducible factor controls the expression of genes in response to hypoxia. It has been reported that the expression of hypoxia-inducible factor-1α protein is an important independent favourable prognostic factor for survival in patients with DLBCL treated with R-CHOP^39^, which indicates hypoxia response pathway plays substantial role in DLBCL relapse on R-CHOP. Further work is needed to understand how DNA methylation changes translate into expression changes at relapse.

We also investigated for the first time the correlation between intra-tumour MH and relapse. We observed decreased intra-tumour MH with tumour evolution, which is consistent with clonal selection process underlying tumour progression after treatment. Intra-tumour MH was first explored by Varley *et al.*^40^ at *MLH1* promoter and was correlated with clinical outcome in lymphomas by De *et al.*^13^. In the latter study, intra-tumour MH was defined by the abundance of intermediate methylation states (not fully methylated nor fully unmethylated) in lymphomas. They concluded that the extent of intra-tumour MH in DNA methylation increases with disease aggressiveness, which is consistent with results shown here^13^ (although they did not study relapsed DLBCLs). A recent paper by Oakes *et al.*^41^ revealed that increasing MH in chronic lymphocytic leukaemia correlates with advanced genetic subclonal complexity. Oakes *et al.*^41^ characterized intra-tumour MH in single CpG sites using an approach called MH, which is calculated by summing all values between 20 and 80% methylation subtracted by the amount of estimated genomic allele specific methylation. Despite the important findings reported by these authors, the approach used to quantify heterogeneity does not take into account adjacent CpG sites, which can only be examined using bisulfite sequencing-based DNA methylation-profiling approaches such as ERRBS. MH analysis from single CpG sites is limited since it is possible to find little variation of DNA methylation at single CpG sites in the context of high intra-tumour MH on the larger locus including adjacent CpGs. For example, in Supplementary Fig. 11, locus A and locus B have identical DNA methylation level (80%) at all four CpG sites. By definition, MH values are 0 for all loci (and all CpGs) in both A and B loci. However, using the epipolymorphism approach, Locus B has higher MH than Locus A. Intuitively, there are more distinct methylation patterns in the cell population of Locus B, which came from different cells (Supplementary Fig. 7). We conclude that the method used in the present study is more sensitive for quantification of intra-tumour MH. We used our intra-tumour MH analysis to characterize MH genome-wide but also within local regions. We found that many regions had no significant DNA methylation changes but significant changes in intra-tumour MH. Such patterns could not be found by previous studies mostly due to use of array-based methylation-profiling techniques.

We also found that intra-tumour MH at diagnosis is lower in patients who relapsed compared with patients who had not relapsed in 5 years after diagnosis. This was shown in two distinct cohorts. This is the first study that used intra-tumour MH at diagnosis specifically as a predictor of relapse occurrence. Patients with lower intra-tumour MH had a longer time to progression compared with patients with highly disordered methylation status, which further supports the important role of methylome evolution in tumour progression. Most importantly, intra-tumour MH does not clearly correlate with genetic clonal heterogeneity measured by VDJ-seq, suggesting that intra-tumour epigenomic heterogeneity does not simply derive from clonal heterogeneity. We investigated potential mechanisms that could underlie epigenomic heterogeneity; for example, we studied MH on *AICDA* short hairpin RNA-mediated knockdown but did not observe any decrease or increase in MH (A.M.M., O.E., R.S., personal communication). Therefore, mechanisms responsible for epigenomic heterogeneity remain unknown and require further investigations. While the use of epigenomic heterogeneity to predict which patients are at higher risk of relapse may have clinical implications, prospective validation studies will help define the true clinical utility of our findings and explain the underlying biological process. Altogether, however, our study firmly indicates that DNA methylation and the epigenome evolve in time and especially on treatment and should be considered alongside genomic evolution when seeking to explain tumour evolution.

## Methods

### DNA extraction

DNA was extracted from frozen solid tissue sections. The tumour purity of those samples were found to be above 80–90% based on histological observation. Frozen tissue samples were first digested overnight with 0.5 mg ml^−1^ Proteinase K and 0.625% SDS in 4 ml Nucleic Lysis Buffer at 37 °C. After digestion, 1 ml of saturated NaCl was added to the samples and samples were shaken vigorously for 15 s before spun at 2,500 r.p.m. for 15 min. Supernatant was transferred to a new tube and mixed with two volumes of room temperature 100% ethanol. DNA was precipitated by centrifugation at maximum speed for 30 min, washed twice with 70% ethanol and finally dissolved in TE or nuclease-free water overnight at room temperature.

### Enhanced reduced representation bisulfite sequencing

Sample preparations were performed at the WCMC Epigenomics Core Facility^14^. In brief, the DNA was digested with MspI enzyme first and then ligated with 5-methylcytosine-containing Illumina adapters. Adaptor ligated DNA fragments were size selected (150–400 bp) and processed with bisulfite conversion using the EZ DNA methylation Kit (Zymo Research, Irvine, CA) as described^42^. Bisulfite-converted DNA was then amplified with Illumina PCR primers PE1.0 and 2.0 for 18 cycles. PCR products were cleaned using Agencourt AMPure XP (Beckman Coulter, Brea, CA) beads as per the manufacturer's recommended protocol (Agencourt) and sequenced on Illumina HiSeq2000. The WCMC Computational Genomics Core Facility supported ERRBS data analysis^14^. In brief, bisulfite reads were aligned to the bisulfite-converted hg19 reference genome using Bismark^43^. All samples had bisulfite conversion rates >99.7%.

### CTCF ChIP-seq in DLBCL OCI-Ly1 cell

In brief, 25 million cells were crosslinked with 1% formaldehyde for 10 min at room temperature. After quenching with 0.125 M glycine for 5 min, cells were washed with PBS twice, and resuspended in Szak RIPA buffer and left on ice for at least 20 min before sonication. After sonication, immunoprecipitations were performed using 5 μg anti-CTCF antibody (Millipore, 07-729) or RbIgG control antibody (Abcam, ab46540). Deep-sequencing libraries were constructed from 10 ng ChIP or Input DNA following Illumina protocol. Final library product (7 pM) was sequenced either on GA_IIX_. Peak calling was performed using ChIPseeqer^22^.

### RNA-seq

Total RNA was extract from patient samples using Trizol (LifeTechnologies). RNA concentration was determined using Qubit (LifeTechnologies) and integrity was verified using Agilent 2100 Bioanalyzer (Agilent Technologies). Libraries were generated using mRNA-seq sample prep kit (Illumina), through which mRNA was selected by two rounds of purification using magnetic polydT beads and then fragmented. First-strand synthesis was performed using random oligos and SuperscriptIII (Invitrogen). After second-strand synthesis, a 200-bp paired-end library was prepared following the Illumina paired-end library preparation protocol. Pair-end sequencing (PE50) was performed on Illumina HiSeq2000.

### MassArray

For validation of DNA methylation measured by ERRBS, single-locus quantitative DNA methylation was performed on bisulfite-converted DNA (EZ DNA Methylation Kit, Zymo Research) using MassArray assay (Sequenom, CA). Primers were designed to cover CpG dense areas of interest by using Sequenom EpiDesigner beta software (http://www.epidesigner.com/).

### Bisulfite-PCR-MiSeq

To validate intra-tumour DNA MH measured by ERRBS, we performed bisulfite-PCR on several loci of interest. Bisulfite conversion was performed using the EZ DNA Methylation Kit from Zymo Research. PCR primers were designed by using Sequenom EpiDesigner beta software. PCR products were gel purified, and then converted to sequencing libraries following Illumina TruSeq protocol. Pair-end sequencing (250 bp either end) was performed on Illumina MiSeq machine. In brief, bisulfite reads were aligned to the bisulife-converted hg19 reference genome using Bismark^43^, with non-directional model.

### Computational approaches for analysing ERRBS data

Differentially methylated regions (DMRs) were defined as regions containing at least five DMCs (false discovery rate=20%, Fisher Exact Tests) and whose total methylation difference was more than 10%. The use of five or more DMCs partially overcomes the statistical limitation of individual Fisher exact tests based on *n*=1 patient samples, as the latter should be cautiously interpreted in the absense of multiple measurements from indepdent samples. DMRs were annotated using ChIPseeqerAnnotate from the ChIPseeqer package^22^. Pathway analyses were performed with default parameters using iPAGE^20^. Methylation of a specific region was calculated by averaging the methylation levels of all covered CpGs in that region.

To determine a methylation signature based on our identified differentially methylated regulatory elements, we identified 107 genes, which had differentially methylated promoters. Considering enhancers can regulated genes at distances reaching 1 Mb (ref. 44), we then identified 369 genes that were located in the neighbourhood of these hypomethylated enhancers (<1 Mb). We did not find hypermethylated enhancers in our cohort. Although CTCF can form loop up to 1 Mb, about 28% CTCF-binding sites located <10 kb from nearest genes. Thus, we identified 67 genes located in the neighbour of differentially methylated CTCF sites (≤10 kb, 17 genes near the hypermethylated sites and 50 genes near the hypomethylated sites).

### GO analysis

The gene ontology (GO) analyses were performed with iPAGE^20^. The concept of mutual information (MI)^45^ to directly quantify the dependency between expression or methylation and known pathways in the GO^46^ or in the lymphoid signature database from the Staudt Lab^21^ are used in iPAGE. Non-parametric statistical tests are then used to determine whether a pathway is significantly informative about the observed methylation measurements. An iPAGE input file is defined across around 24,000 genes from Refseq genes, where each gene is associated with a unique methylation status in our analysis. In the analysis of the diagnosis–relapse signature (Fig. 3a,b), each gene is either stable, hyper- or hypomethylated genes from diagnosis to relapse. In the heterogeneity analysis (Fig. 5b), each gene is associated with either stable or decreased heterogeneity in another GO analysis). Meanwhile, each gene can be associated with a subset of *M* known pathways (for example, from the GO annotations). For each pathway, the pathway profile is defined as binary vector with *N* elements, one for each gene. ‘1' indicates that the gene belongs to the pathway and ‘0' indicates that it does not.

Given a pathway profile and a methylation file with *N*_e_ groups, iPAGE creates a table *C* of dimensions 2 × *N*_e_, in which *C*(1, *j*) represents the number of genes that are contained in the *j*^th^ methylation group and are also present in the given pathway. *C*(2,*j*) contains the number of genes that are in the *j*^th^ methylation group but not assigned to the pathway. Given this table, we calculate the empirical MI as follows:


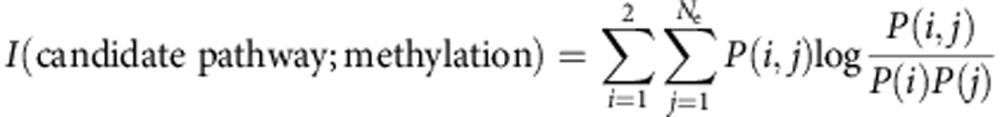


where


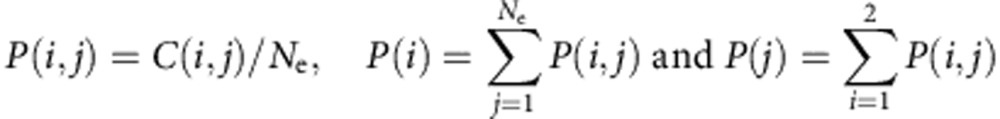


To assess the statistical significance of the calculated MI values, we used a non-parametric randomized-based statistical test. Given *I* as the real MI value and keeping the pathway profile unaltered, the methylation file is shuffled 10,000 times and the corresponding MI values *I*_random_ are calculated. A pathway is accepted only if *I* is larger than (1-*max_p*) of the *I*_random_ values (*max_p* is set to 0.005). This corresponds to a *P* value <0.005. In iPAGE, pathways are first sorted by information (from informative to non-informative). Starting from the most informative pathways, the statistical test described above is applied to each pathway, and pathways that pass the test are returned. When 20 contiguous pathways in the sorted list do not pass the test, the procedure is stopped.

Highly statistically significant MI is explained by the combination of over-representation and under-representation in specific methylation groups. To quantify the level of over- and under-representation, the hypergeometric distribution is used to calculate two distinct *P* values:

(a) For over-representation:


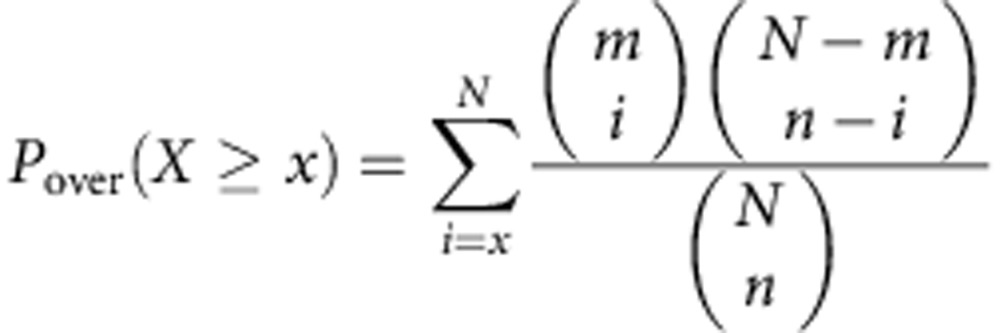


(b) For under-representation:


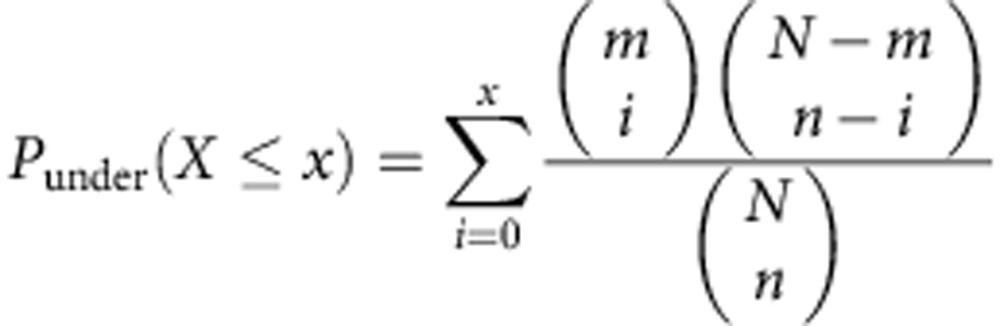


where *x* equals the number of genes in the given methylation group, which are also assigned to the give pathway. *m* is the number of genes assigned to the pathway (foreground), *n* is the number of genes in the methylation group and *N* is the total number of genes (background). If *p*_over_<*p*_under_, we consider the pathway to be over-represented in the methylation cluster; otherwise, it is under-represented. In the heatmap such as those in Figs 3a,b and 5b, colours indicate over- or under-representation levels. The red colour indicates (in log_10_) the over-represented *P* values and the blue shows under-representation.

### Intra-tumour MH calculation

Methylation epipolymorphism was calculated as defined in Landan's paper^30^. The epipolymorphism level of a 4-CpG locus in the cell population was defined as the probability that epialleles randomly sampled from the locus differ from each other. More specifically, if we denote *p*_*i*_ as for the fraction of each DNA methylation pattern *i* in the cell population. The epipolymorphism equals 1—*Σp*_*i*_^*2*^. The higher the epipolymorphism, the higher the intra-tumour heterogeneity is. Since the epipolymorphism levels are dependent on DNA methylation levels, we binned all loci based on average methylation level into 21 bins spanning 0–100% methylation levels. All the loci in one sample were divided into 21 different groups according to their average methylation level. The width of each bin is 5 except the first and last one ((0%, 2.5%) and (97.5%, 100%)). We then calculated the median epipolymorphism in each group. Median epipolymorphism across all groups defined the overall epipolymorphism landscape across the spectrum of methylation levels. The intra-tumour MH was calculated by determining the area under median epipolymorphism line. The area under median line by obtained by summing up median epipolymorphism value across all 21 bins weighted by the width of the bin. Intra-tumour MH scales from 0 to 100. Larger areas mean higher intra-tumour heterogeneity. Two analyses were conducted. In the first one (CGIs), all loci were selected based on the following standards: (i) location within CGIs; (ii) covered by more than 60 reads; (iii) the largest distance between the first and the fourth CpG sites was 9 bp.The number of such loci was about 20,000 on average when using our ERRBS platform. For each locus of four CpGs, we counted how many times each of the 16 possible methylation states was observed, obtaining statistics on the distribution of DNA methylation patterns at thousands of loci. In the second one (promoters), promoter loci were selected as follows: (i) all loci were located in gene promoter regions (defined as ±2 kb windows centred on RefSeq transcription start sites); (ii) each locus was covered by more than 60 reads. We obtained about 300,000 loci in each sample (10-fold more than the previous analysis) for this analysis from ERRBS. For Bisulfite-PCR-MiSeq data, we obtained around 170 loci in each sample from targeted regions. The code for performing these analyses will be made available on request.

## Author contributions

H.P. and D.R. analysed the data. Y.J., K.N. and R.S. performed the experiments. G.G.I., W.T., M.B., F.T., L.C., R.S., A.M.M., M.L. and A.C. provided the clinical samples and reagents. H.P., Y.J., A.M.M., G.G.I., W.T. and O.E. wrote manuscript. W.T. and O.E. conceived and supervised the study.

## Additional information

**Accession codes:** The data discussed in this publication have been deposited in NCBI's Gene Expression Omnibus and are accessible through GEO Series accession number GSE66329.

**How to cite this article:** Pan, H. *et al.* Epigenomic evolution in diffuse large B-cell lymphomas. *Nat. Commun.* 6:6921 doi: 10.1038/ncomms7921 (2015).

## Supplementary Material

Supplementary Information Supplementary Figures 1-11, Supplementary Note 1 and Supplementary References

Supplementary Data 1 Clinical information for patients in cohort 1

Supplementary Data 2 Number of CpGs with more than 10X coverage for patients in cohort 1 and cohort 2

Supplementary Data 3 Number of DMCs and DMRs between diagnostic and relapsed patient sample pairs

Supplementary Data 4 Top 1000 DMCs from patient 1.1

Supplementary Data 5 Top 1000 DMRs from patient 1.1

Supplementary Data 6 Differentially methylated regulatory elements between diagnostic and relapsed sample pairs

Supplementary Data 7 Average DNA methylation correlation between ERRBS and MassArray

Supplementary Data 8 Epipolymorphism difference for all the methylation bins from all the sample pairs

Supplementary Data 9 Average epipolymorphism correlation between ERRBS and MiSeq

Supplementary Data 10 Clinical information for patients in cohort 2

## References

[b1] Armitage J. O. & Weisenburger D. D. New approach to classifying non-Hodgkin's lymphomas: clinical features of the major histologic subtypes. J. Clin. Oncol. 16, 2780–2795 (1998). 9704731 10.1200/JCO.1998.16.8.2780

[b2] Morton L. M. *et al.* Lymphoma incidence patterns by WHO subtype in the United States, 1992-2001. Blood 107, 265–276 (2006). 16150940 10.1182/blood-2005-06-2508 PMC1895348

[b3] Coiffier B. *et al.* CHOP chemotherapy plus rituximab compared with CHOP alone in elderly patients with diffuse large-B-cell lymphoma. N. Engl. J. Med. 346, 235–242 (2002). 11807147 10.1056/NEJMoa011795

[b4] Larouche J.-F. *et al.* Lymphoma recurrence 5 years or later following diffuse large B-cell lymphoma: clinical characteristics and outcome. J. Clin. Oncol. 28, 2094–2100 (2010). 20308668 10.1200/JCO.2009.24.5860

[b5] Abramson J. S. & Shipp M. A. Advances in the biology and therapy of diffuse large B-cell lymphoma: moving toward a molecularly targeted approach. Blood 106, 1164–1174 (2005). 15855278 10.1182/blood-2005-02-0687

[b6] Hunt K. E. & Reichard K. K. Diffuse large B-cell lymphoma. Arch. Pathol. Lab. Med. 132, 118–124 (2008). 18181663 10.5858/2008-132-118-DLBL

[b7] Shaknovich R. & Melnick A. Epigenetics and B-cell lymphoma. Curr. Opin. Hematol. 18, 293–299 (2011). 21577103 10.1097/MOH.0b013e32834788cf PMC3260081

[b8] Berdasco M. & Esteller M. Aberrant epigenetic landscape in cancer: how cellular identity goes awry. Dev. Cell 19, 698–711 (2010). 21074720 10.1016/j.devcel.2010.10.005

[b9] Lai A. Y. *et al.* DNA methylation prevents CTCF-mediated silencing of the oncogene BCL6 in B cell lymphomas. J. Exp. Med. 207, 1939–1950 (2010). 20733034 10.1084/jem.20100204 PMC2931164

[b10] Hogan L. E. *et al.* Integrated genomic analysis of relapsed childhood acute lymphoblastic leukemia reveals therapeutic strategies. Blood 118, 5218–5226 (2011). 21921043 10.1182/blood-2011-04-345595 PMC3217405

[b11] Feng S., Kroll M. H., Nolasco L., Moake J. & Afshar-Kharghan V. Genome-wide DNA methylation profiling predicts relapse in childhood B-cell acute lymphoblastic leukaemia. Br. J. Haematol. 160, 404–406 (2013). 23110451 10.1111/bjh.12113 PMC3568176

[b12] Clozel T. *et al.* Mechanism-based epigenetic chemosensitization therapy of diffuse large B-cell lymphoma. Cancer Discov. 3, 1002–1019 (2013). 23955273 10.1158/2159-8290.CD-13-0117 PMC3770813

[b13] De S. *et al.* Aberration in DNA methylation in B-cell lymphomas has a complex origin and increases with disease severity. PLoS Genet. 9, e1003137 (2013). 23326238 10.1371/journal.pgen.1003137 PMC3542081

[b14] Akalin A. *et al.* Base-pair resolution DNA methylation sequencing reveals profoundly divergent epigenetic landscapes in acute myeloid leukemia. PLoS Genet. 8, e1002781 (2012). 22737091 10.1371/journal.pgen.1002781 PMC3380828

[b15] Shaknovich R. *et al.* DNA methyltransferase 1 and DNA methylation patterning contribute to germinal center B-cell differentiation. Blood 118, 3559–3569 (2011). 21828137 10.1182/blood-2011-06-357996 PMC3186332

[b16] Chambwe N. *et al.* Variability in DNA methylation defines novel epigenetic subgroups of DLBCL associated with different clinical outcomes. Blood 123, 1699–1708 (2014). 24385541 10.1182/blood-2013-07-509885 PMC3954051

[b17] Lai A. Y. *et al.* DNA methylation profiling in human B cells reveals immune regulatory elements and epigenetic plasticity at Alu elements during B-cell activation. Genome Res. 23, 2030–2041 (2013). 24013550 10.1101/gr.155473.113 PMC3847773

[b18] Shaknovich R. *et al.* DNA methylation signatures define molecular subtypes of diffuse large B-cell lymphoma. Blood 116, e81–e89 (2010). 20610814 10.1182/blood-2010-05-285320 PMC2993635

[b19] Lin P.-C. C. *et al.* Epigenomic alterations in localized and advanced prostate cancer. Neoplasia 15, 373–383 (2013). 23555183 10.1593/neo.122146 PMC3612910

[b20] Goodarzi H., Elemento O. & Tavazoie S. Revealing global regulatory perturbations across human cancers. Mol. Cell 36, 900–911 (2009). 20005852 10.1016/j.molcel.2009.11.016 PMC2900319

[b21] Shaffer A. L. *et al.* A library of gene expression signatures to illuminate normal and pathological lymphoid biology. Immunol. Rev. 210, 67–85 (2006). 16623765 10.1111/j.0105-2896.2006.00373.x

[b22] Giannopoulou E. G. & Elemento O. An integrated ChIP-seq analysis platform with customizable workflows. BMC Bioinformatics 12, 277 (2011). 21736739 10.1186/1471-2105-12-277 PMC3145611

[b23] Hatzi K. *et al.* A hybrid mechanism of action for BCL6 in B cells defined by formation of functionally distinct complexes at enhancers and promoters. Cell Rep. 4, 578–588 (2013). 23911289 10.1016/j.celrep.2013.06.016 PMC3854650

[b24] Aran D., Sabato S. & Hellman A. DNA methylation of distal regulatory sites characterizes dysregulation of cancer genes. Genome Biol. 14, R21 (2013). 23497655 10.1186/gb-2013-14-3-r21 PMC4053839

[b25] Bell A. C. & Felsenfeld G. Methylation of a CTCF-dependent boundary controls imprinted expression of the Igf2 gene. Nature 405, 482–485 (2000). 10839546 10.1038/35013100

[b26] Schoenherr C. J., Levorse J. M. & Tilghman S. M. CTCF maintains differential methylation at the Igf2/H19 locus. Nat. Genet. 33, 66–69 (2003). 12461525 10.1038/ng1057

[b27] Rai D., Kim S.-W., McKeller M. R., Dahia P. L. M. & Aguiar R. C. T. Targeting of SMAD5 links microRNA-155 to the TGF-beta pathway and lymphomagenesis. Proc. Natl Acad. Sci. USA 107, 3111–3116 (2010). 20133617 10.1073/pnas.0910667107 PMC2840369

[b28] Feng X.-H. & Derynck R. Specificity and versatility in tgf-beta signaling through Smads. Annu. Rev. Cell Dev. Biol. 21, 659–693 (2005). 16212511 10.1146/annurev.cellbio.21.022404.142018

[b29] Bakkebø M., Huse K., Hilden V. I., Smeland E. B. & Oksvold M. P. TGF-β-induced growth inhibition in B-cell lymphoma correlates with Smad1/5 signalling and constitutively active p38 MAPK. BMC Immunol. 11, 57 (2010). 21092277 10.1186/1471-2172-11-57 PMC3006362

[b30] Landan G. *et al.* Epigenetic polymorphism and the stochastic formation of differentially methylated regions in normal and cancerous tissues. Nat. Genet. 44, 1207–1214 (2012). 23064413 10.1038/ng.2442

[b31] Landau D. A. *et al.* Locally disordered methylation forms the basis of intratumor methylome variation in chronic lymphocytic leukemia. Cancer Cell 26, 813–825 (2014). 25490447 10.1016/j.ccell.2014.10.012 PMC4302418

[b32] Jiang Y. *et al.* Deep-sequencing reveals clonal evolution patterns and mutation events associated with relapse in B cell lymphomas. Genome Biol. 15, 432 (2013). 25123191 10.1186/s13059-014-0432-0 PMC4158101

[b33] Lenz G. & Staudt L. M. Aggressive lymphomas. N. Engl. J. Med. 362, 1417–1429 (2010). 20393178 10.1056/NEJMra0807082 PMC7316377

[b34] Hartmann E. M., Ott G. & Rosenwald A. Molecular biology and genetics of lymphomas. Hematol. Oncol. Clin. North Am. 22, 807–823 vii (2008). 18954738 10.1016/j.hoc.2008.07.004

[b35] Carroll S. B. Evo-devo and an expanding evolutionary synthesis: a genetic theory of morphological evolution. Cell 134, 25–36 (2008). 18614008 10.1016/j.cell.2008.06.030

[b36] Ngo V. N. *et al.* A loss-of-function RNA interference screen for molecular targets in cancer. Nature 441, 106–110 (2006). 16572121 10.1038/nature04687

[b37] Iqbal J. *et al.* BCL2 expression is a prognostic marker for the activated B-cell-like type of diffuse large B-cell lymphoma. J. Clin. Oncol. 24, 961–968 (2006). 16418494 10.1200/JCO.2005.03.4264

[b38] Molina-Privado I. *et al.* E2F4 plays a key role in Burkitt lymphoma tumorigenesis. Leukaemia 26, 2277–2285 (2012). 10.1038/leu.2012.99 22475873

[b39] Evens A. M. *et al.* Hypoxia-inducible factor-1 {alpha} expression predicts superior survival in patients with diffuse large B-cell lymphoma treated with R-CHOP. J. Clin. Oncol. 28, 1017–1024 (2010). 20048181 10.1200/JCO.2009.24.1893 PMC2834428

[b40] Varley K. E., Mutch D. G., Edmonston T. B., Goodfellow P. J. & Mitra R. D. Intra-tumor heterogeneity of MLH1 promoter methylation revealed by deep single molecule bisulfite sequencing. Nucleic Acids Res. 37, 4603–4612 (2009). 19494183 10.1093/nar/gkp457 PMC2724279

[b41] Oakes C. C. *et al.* Evolution of DNA methylation is linked to genetic aberrations in chronic lymphocytic leukemia. Cancer Discov. 4, 348–361 (2013). 24356097 10.1158/2159-8290.CD-13-0349 PMC4134522

[b42] Will B. *et al.* Satb1 regulates the self-renewal of hematopoietic stem cells by promoting quiescence and repressing differentiation commitment. Nat. Immunol. 14, 437–445 (2013). 23563689 10.1038/ni.2572 PMC3633104

[b43] Krueger F. & Andrews S. R. Bismark: a flexible aligner and methylation caller for bisulfite-seq applications. Bioinformatics 27, 1571–1572 (2011). 21493656 10.1093/bioinformatics/btr167 PMC3102221

[b44] Lettice L. A. A long-range Shh enhancer regulates expression in the developing limb and fin and is associated with preaxial polydactyly. Hum. Mol. Genet 12, 1725–1735 (2003). 12837695 10.1093/hmg/ddg180

[b45] Cover T. M. & Thomas J. A. Elements of Information Theory. Elements of Information Theory John Wiley & Sons, Inc. (2006).

[b46] Ashburner M. *et al.* Gene ontology: tool for the unification of biology. The Gene Ontology Consortium. Nat. Genet. 25, 25–29 (2000). 10802651 10.1038/75556 PMC3037419

