# Supplementary material for: Epigenomic evolution in diffuse large B-cell lymphomas
Source: Nat Commun. 2015 Apr 20;6:6921. doi: 10.1038/ncomms7921 (PMC4411286; doi:10.1038/ncomms7921)
Supplement: Supplementary Information — Supplementary Figures 1-11, Supplementary Note 1 and Supplementary References [file ncomms7921-s1.pdf]

SUPPLEMENTARY FIGURES

Supplementary Figure 1

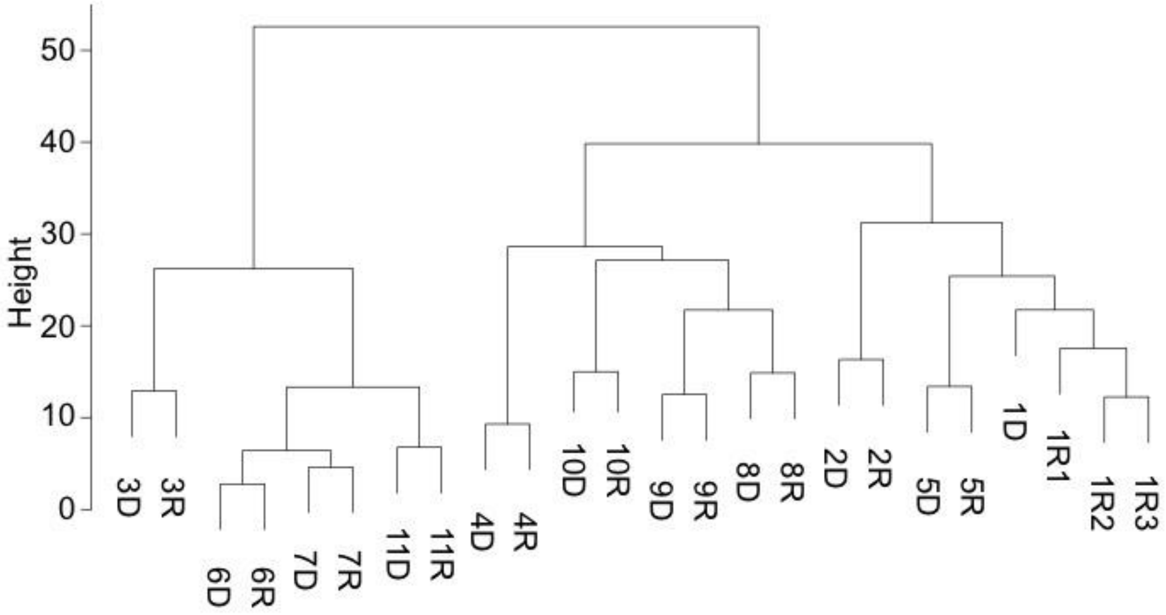

Supplementary Figure 1. Hierarchical clustering of CGI methylation levels in all diagnosis-relapse pairs. Cluster analysis was performed using squared euclidean distance and complete linkage clustering.

Supplementary Figure 2

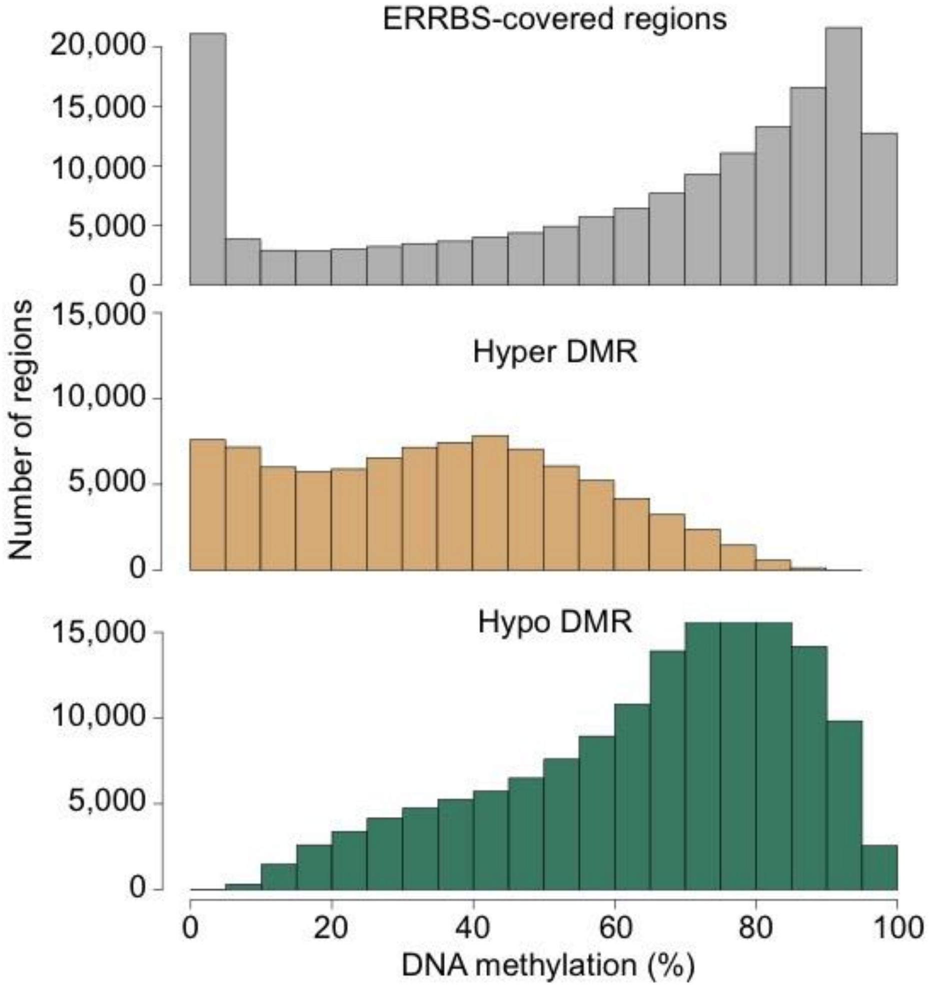

Supplementary Figure 2. Average DNA methylation levels of Hypermethylation, Hypomethylation and ERRBS-covered regions, at diagnosis. DNA methylation level of specific region was calculated by the average DNA methylation of all the CpGs in the corresponding region. Figures indicate the number of regions with different average DNA methylation levels. Top, middle and bottom panels represent ERRBS-covered regions, Hyper DMRs and Hypo DMRs. The analysis combined data from 11 diagnosis samples in Cohort 1.

Supplementary Figure 3

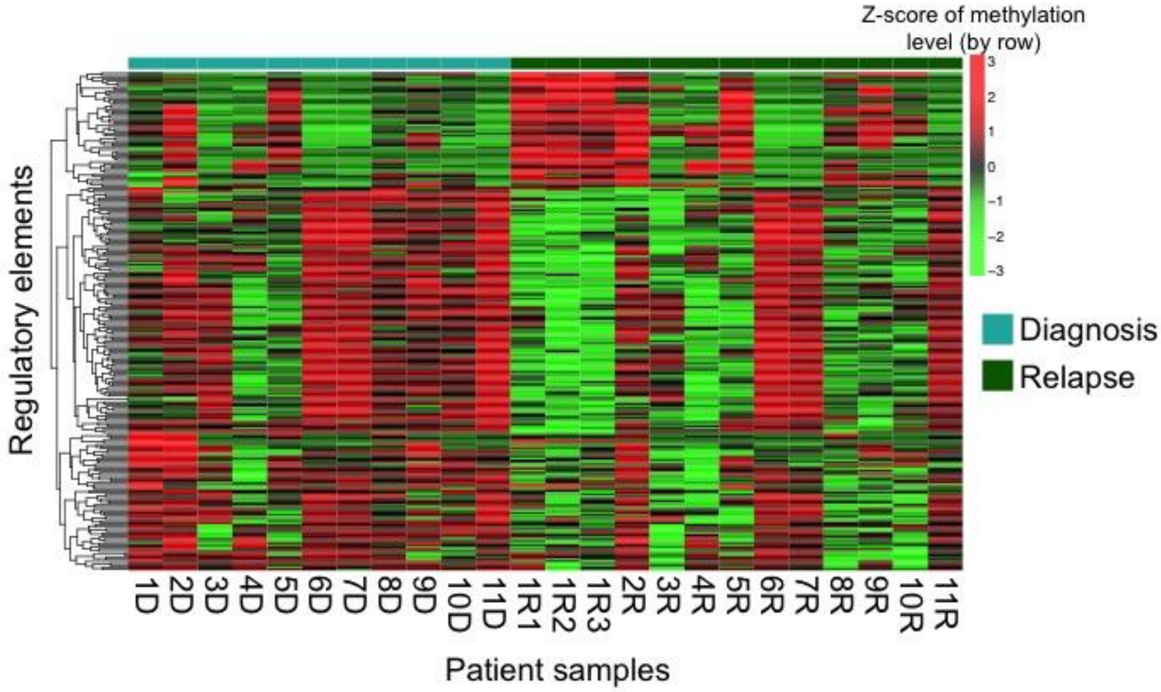

Supplementary Figure 3. Heat map of DNA methylation levels of regulatory elements. Each row represent single differentially methylated regulatory element. Each column represent single diagnosis/relapsed sample from patients. Scale bar represent z-score of methylation level. Values were centered and scaled in row direction.

Supplementary Figure 4

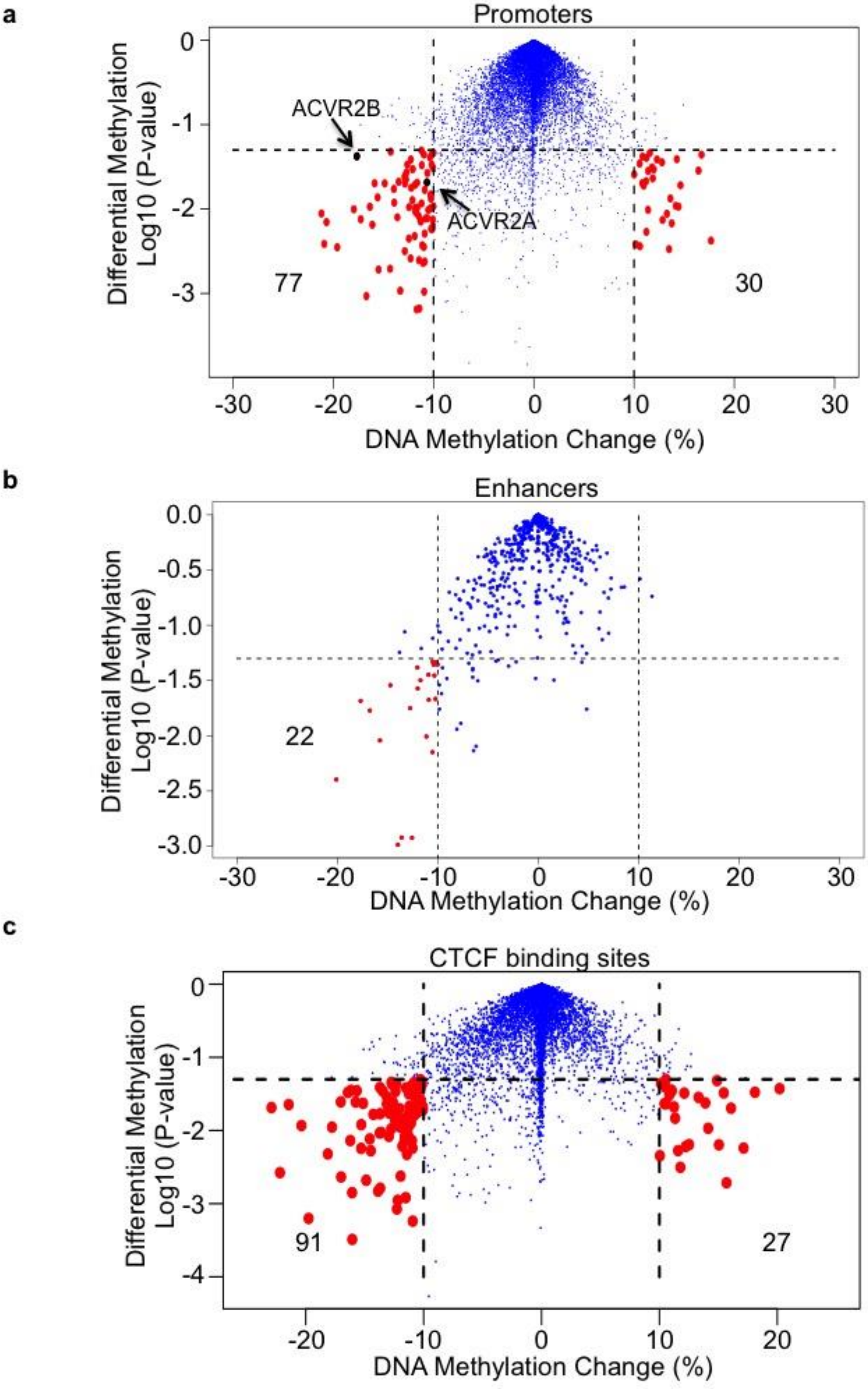

Supplementary Figure 4. Consistently differentially methylated regulatory elements. Figure **a**-**c** represent differentially methylated promoters, enhancers and CTCF binding sites. The DNA methylation changes were calculated by the difference between diagnosis and relapsed samples of the element. Positive values mean hypermethylation at relapse and negative values indicate hypomethylation. P-values came from paired t-test between methylation levels of diagnosis and relapsed samples. Red dots indicate significantly differentially methylated regulatory elements (>10% DNA methylation changes and P<0.05). Numbers in the figure represent the numbers of hyper/hypo differentially methylated regions.

Supplementary Figure 5

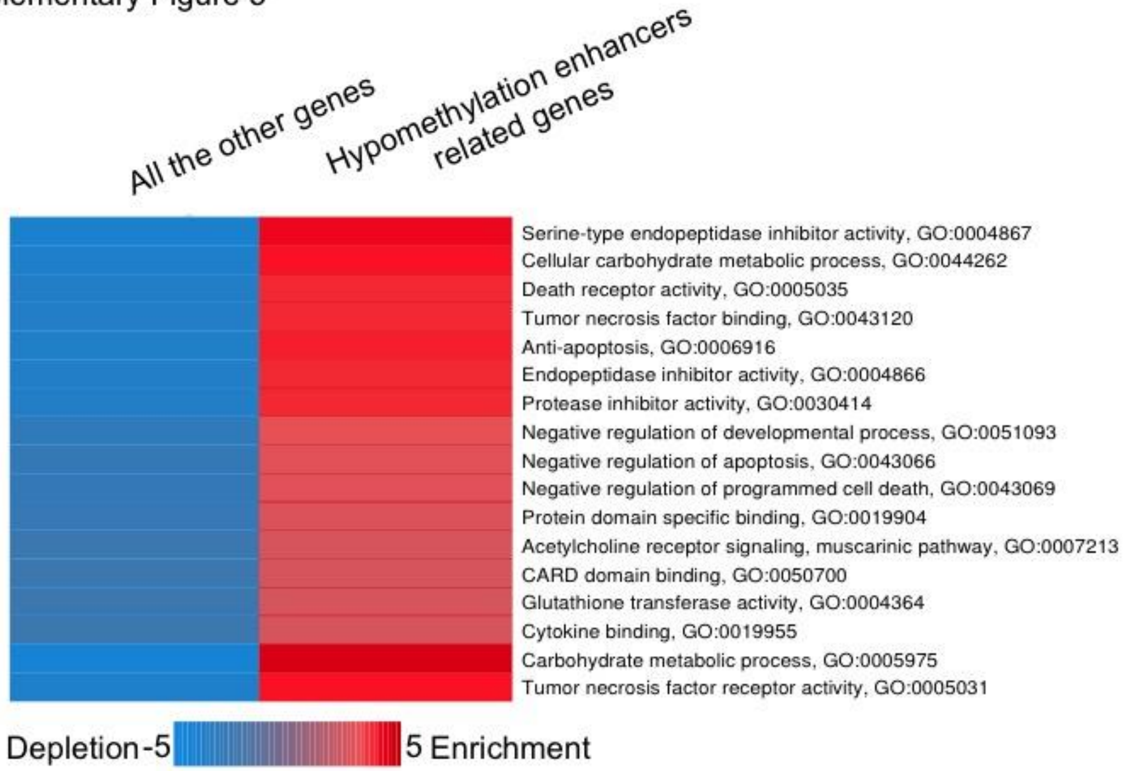

Supplementary Figure 5. Genes near hypomethylated enhancers involved key pathways. Pathways over-represented with genes in the neighborhood of hypomethylation enhancers (<= 1Mb) were illustrated here. GO analyses were performed with iPAGE[1]. Known pathways in the Gene Ontology[2] were used here. The background included around 24,000 genes from Refseq genes. The red color indicates (in $\log_{10}$) the over-represented *p*-values and the blue shows under-representation. P-value were obtained from hypergeometric tests.

Supplementary Figure 6

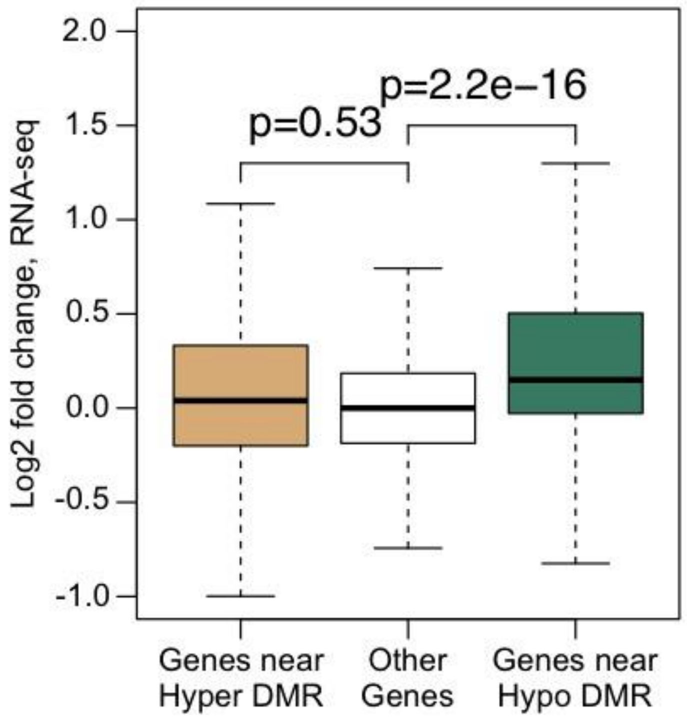

Supplementary Figure 6. Expression fold changes of nearby genes in regard of hyper/hypo methylated DMRs (Patient 2). Hyper/hypo methylation related genes were defined as genes whose promoter is located nearby a DMR (within 5kb). The log2 fold change was calculated by the RPKM value change from diagnosis to relapse in this patient. We performed t-test comparing the expression changes of hyper/hypo DMRs related genes versus expression changes of other genes. P-value were derived from t-test.

Supplementary Figure 7

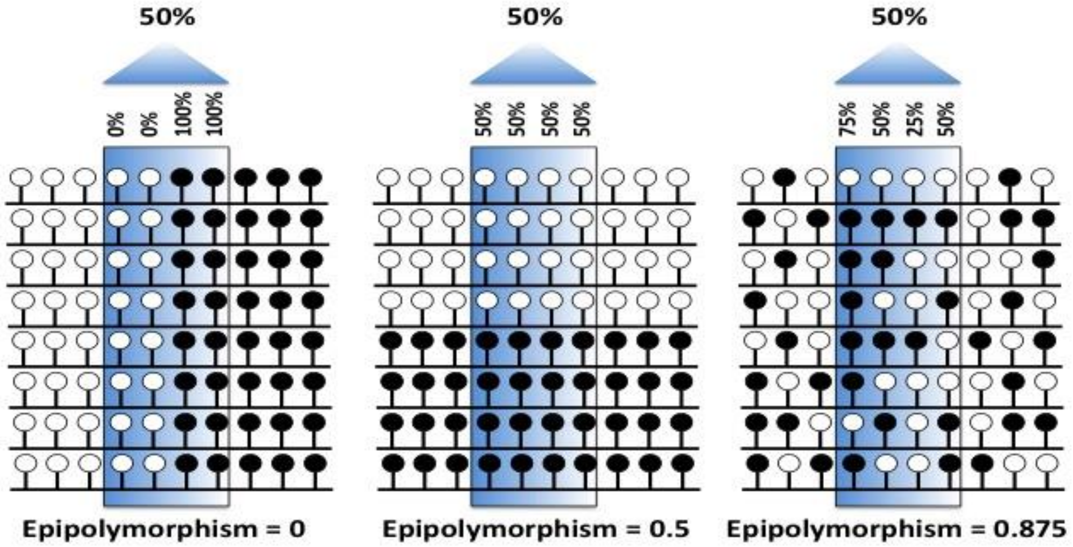

Supplementary Figure 7. Schematic of the DNA methylation patterns at loci with identical average methylation level but different intra-tumor methylation heterogeneity. Empty and filled circles represent unmethylated and methylated CpGs separately. A locus was defined as four adjacent CpGs (indicated by blue box). The epipolymorphism of a locus was defined as the probability two randomly sampled DNA reads differ from each other.

Supplementary Figure 8

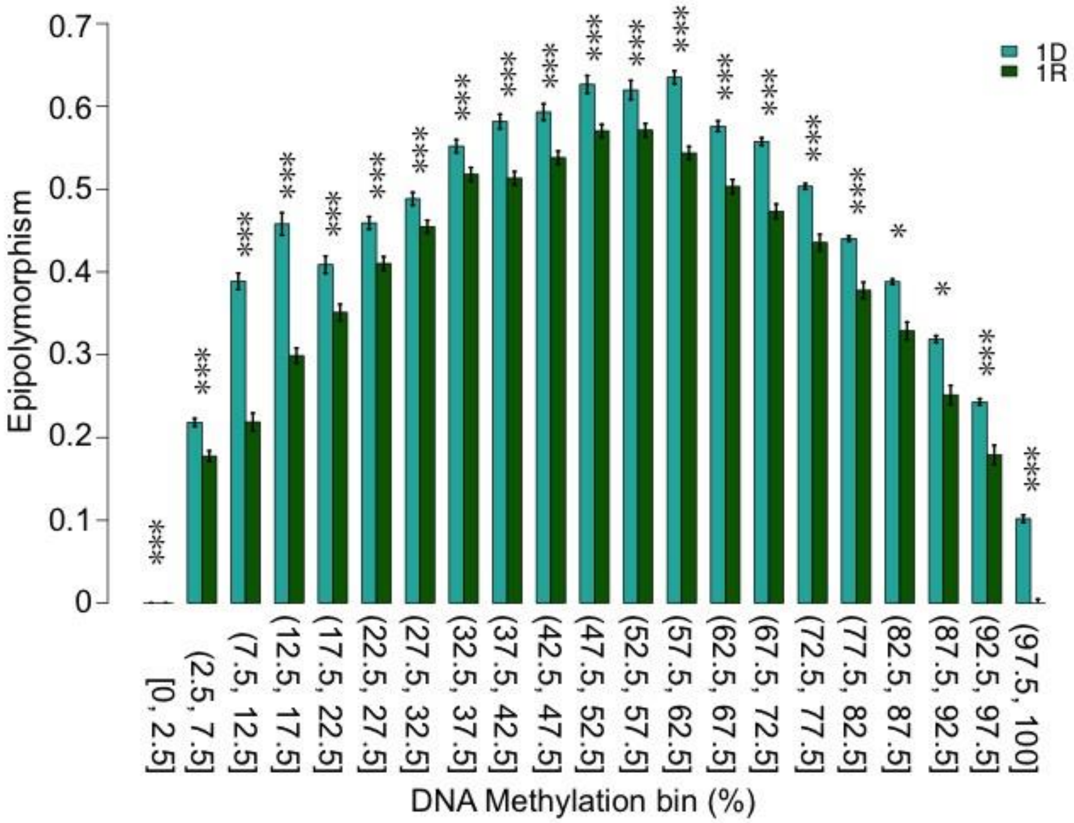

Supplementary Figure 8. Decreased epipolymorphim from diagnosis to relapsed samples in all methylation level bins illustrated in Patient 1.1. Loci from diagnosis and relapsed samples at CGIs were divided into 21 bins based on their methylation levels. Barplot indicates the median epipolymorphism of each bin from diagnosis and relapsed samples. Error bars represent standard errors. Two-sided t-test was used to detect the difference of epipolymorphism on each bin.

*** indicates P-adjust < 0.001

* indicates P-adjust < 0.05

Supplementary Figure 9

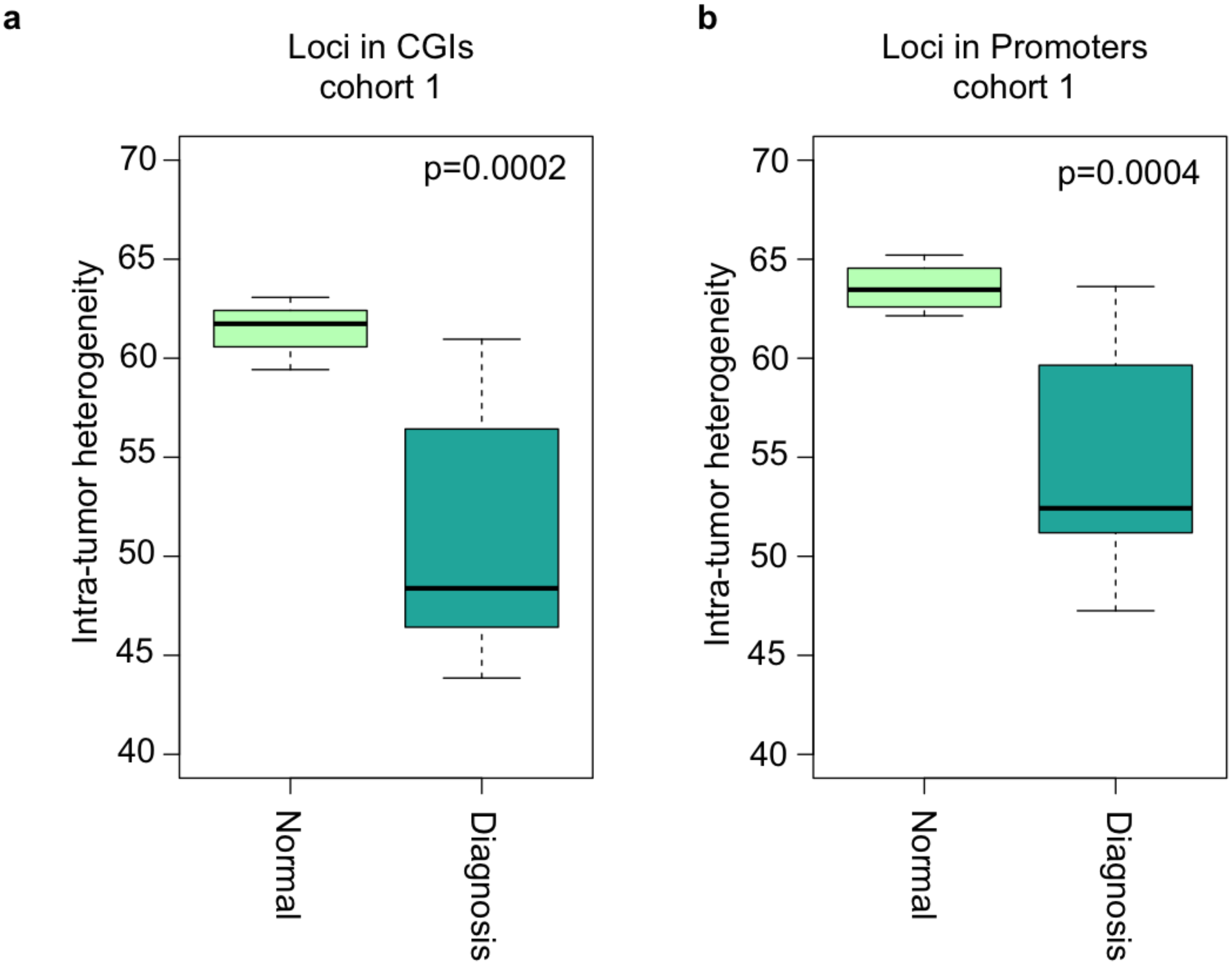

Supplementary Figure 9. Intra-tumor methylation heterogeneity decreases from normal tissues to diagnostic samples. (**a**) Diagnostic samples (n=11) had lower intra-tumor methylation heterogeneity compared to normal tissues (n=4) (Cohort 1). All the loci analyzed were located in CGIs. (**b**) Diagnostic samples (n=11) had lower intra-tumor methylation heterogeneity compared to normal tissues (n=4) (Cohort 1). All the loci analyzed were located in CGIs. All the loci located in promoters. P-values derived from t-test.

Supplementary Figure 10

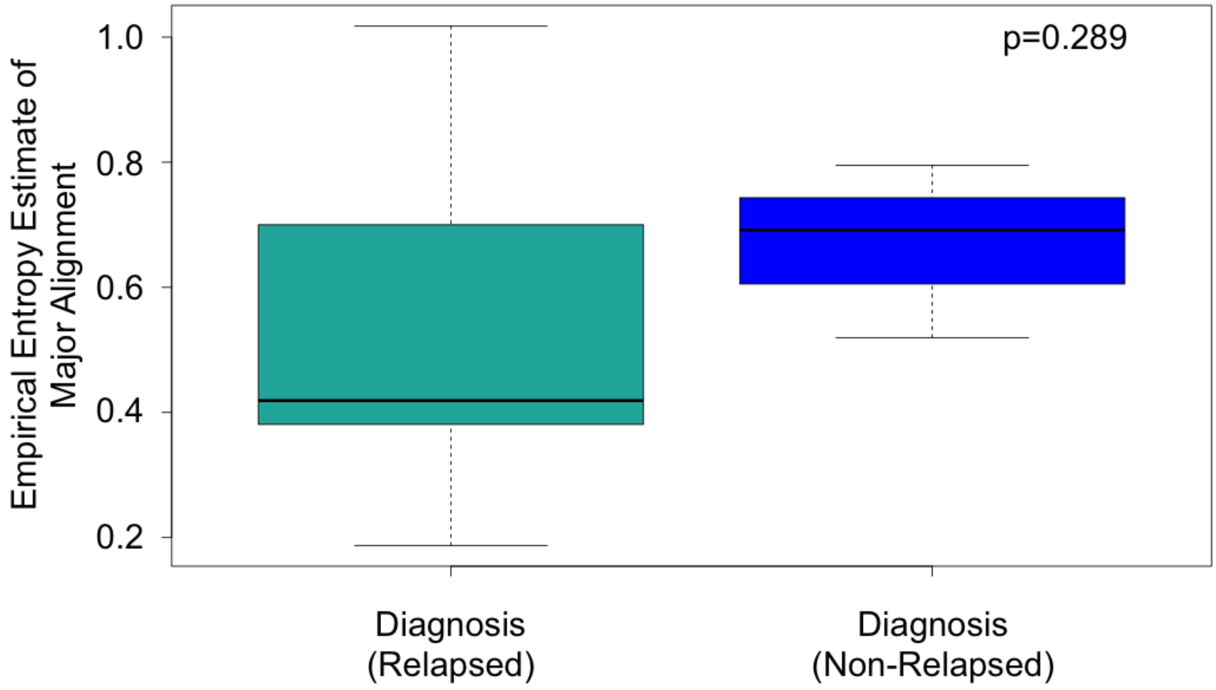

Supplementary Figure 10. Intra-tumor clonal heterogeneity of diagnosis samples from relapsed (N=8) and non-relapsed (N=3) patients. Intra-tumor clonal heterogeneity was measured by the empirical entropy of clonal frequencies of VDJ associated with the main V, D, J rearrangement in each sample. The empirical entropy was calculated by the frequencies of VDJ somatic hypermutation patterns in the tumor population in each patient. P-value was derived from t-test.

Supplementary Figure 11

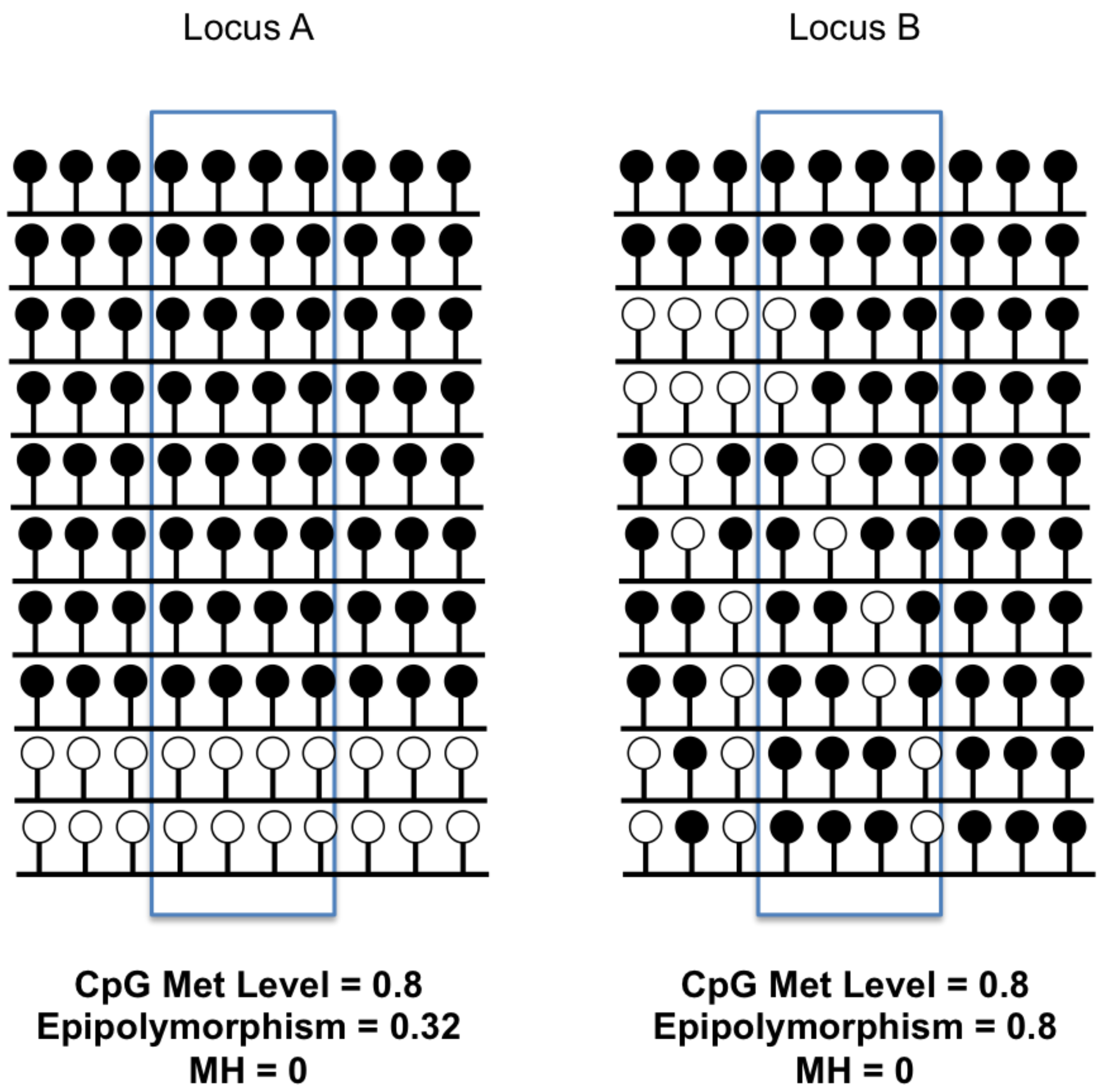

Supplementary Figure 11. **Schematic of locus with identical MH value but distinct methylation patterns.** Each row is a read from a single cell in tumor population. Empty and filled circles represent unmethylated and methylated CpGs. MH was calculated by summing all values between 20-80% methylation subtracted by the amount of estimated genomic ASM[3].

SUPPLEMENTARY NOTE 1

The tendency toward hypo or hyper methylation isn't affected by the distribution of methylation sites in the genome or by the bias of the ERRBS assay toward CG rich regions.

We primarily addressed differences between diagnosis and relapse, which are different (not as strong) as those between normal cells (with marked tendency towards very low or very high and homogeneous methylation) and tumors (with more heterogeneity). We plotted the distribution of methylation levels at diagnosis for hypermethylation and hypomethylation DMRs (**Supplementary Fig. 2**). We also plotted the distribution of methylation levels at diagnosis for all ERRBS-covered regions (**Supplementary Fig. 2**). These regions were defined as containing at least 5 covered CpGs minimum, separated by less than 250bp between contiguous CpGs, which is similar to the definition of DMRs without methylation ratio cutoff and DMCs requirement. We found that (1) DMR methylation levels at diagnosis span across a broad range (not only extreme hypo or hyper methylation) and (2) DMR methylation levels at diagnosis are not well correlated with ERRBS-covered regions. To answer the reviewer's question, these results suggest that (1) the tendency toward hypo or hyper methylation is not strongly affected by the distribution of low and high methylation sites in the genome and (2) the tendency toward hypo or hyper methylation is not strongly affected by the bias of the ERRBS assay toward CG rich regions.

Methylation signature based on differentially methylated regulatory elements

We identified 107 differentially methylated promoters, 22 enhancers and 118 CTCF binding sites between diagnosis and relapse (>10% DNA methylation changes and P<0.05, paired t-test, two-sided) (**Supplementary Fig. 3**, **Supplementary Fig. 4a-c** and **Supplementary data 6**). Next, we associated differentially methylated regulatory elements with genes based on genomic proximity (see **Methods**). In total, we determined 44 hypermethylation related genes and 490 hypomethylation related ones. Genes near hypermethylated regulatory elements included *ADAL, ADAMTS12, AEN, ANO3, ATRNL1, B4GALNT1, C17orf82, C3orf39, CACNA1C, CLSTN2, CRYGN, CXCL12, DDX25, DOCK3, DUSP23, EMX2, FAM171A1, FAM19A5, FNBP1L, FSTL4, GNAS, HCN4, HMCN1, IL1RAPL2, ITGA11, KIAA1217, KLHL34, LCE3A, LCE3B, NKAIN2, NTRK3, OTUB2, PTPRE, RASL10A, RAX, RNF150, RNF157, RORB, SLC25A48, SLC26A10, SRRM3, TTC18, VASH1, WNT5A*. Genes near hypomethylated regulatory elements include *AATK, ABL1, ABTB1, ACAP3, ACP1, ACTG1, ACTL6B, ACVR2A, ACVR2B, ADAM10, ADAMTS17, ADRA2B, AGRN, AIF1L, AKAP1, ALKBH7, ALS2, ALS2CR11, ALS2CR12, AMZ1, ANAPC11, ANKRD34B, AOX1, AQP6, ARHGDIA, ARHGDIG, ARID3C, ARL16, ASS1, ATAD3A, ATAD3B, ATAD3C, ATP2A1, AURKAIP1, AUTS2, AZI1, B3GALT6, BAHCC1, BAIAP2, BCL2, BNIP2, BRAT1, BZRAP1, BZW1, C10orf11, C11orf16, C12orf43, C15orf52, C16orf91, C17orf56, C17orf67, C17orf70, C17orf89, C17orf90, C18orf55, C18orf63,*

C19orf53, C1orf159, C1orf70, C1orf86, C1orf93, C20orf173, C22orf26, C22orf40, C2orf53, C3orf18, C3orf27, C6orf132, C8orf73, C9orf100, C9orf128, C9orf131, C9orf171, C9orf23, CA9, CALML6, CAPN10, CARD11, CARD14, CASP10, CASP8, CBS, CBX2, CBX4, CBX8, CCDC107, CCDC137, CCDC154, CCDC40, CCIN, CCL19, CCL21, CCL27, CCNB2, CCNL2, CD72, CDAN1, CDK11A, CDK11B, CDK15, CELSR1, CEP104, CERK, CERS1, CFLAR, CGB2, CGB8, CHMP6, CHST12, CHST15, CLDN9, CLK1, CLTA, CMYA5, CNDP1, CNDP2, COIL, CPSF3L, CPXM2, CREB3, CREB3L1, CSAG1, CSTL1, CTBP2, CTPS, CUEDC1, CYB5A, CYP20A1, CYP4F11, DCTN3, DCTN6, DFFB, DGKE, DHFR, DIP2C, DNAJB5, DNAJB8, DPM2, DVL1, DYNLL2, DYSFIP1, E2F4, EEFSEC, EFHC1, EIF3B, EIF4A3, ELFN1, ELMO3, EMR1, ENDOV, EPC2, EPX, EXOC3L1, EXOSC2, FAM110C, FAM126B, FAM132A, FAM150B, FAM151B, FAM166B, FAM175B, FAM187B, FAM195A, FAM195B, FAM205A, FAM53B, FAM63B, FAM69C, FAM78A, FAM81A, FANCG, FBXO15, FBXO9, FCAR, FGF17, FOXB1, FSCN2, FTSJ2, FUBP3, FZD7, GAA, GABRD, GALR3, GALT, GATA2, GBA2, GCGR, GCM1, GCNT3, GJC1, GLIPR2, GLTPD1, GNA12, GNB1, GNE, GPR182, GPR26, GPR77, GPR84, GRAMD4, GRK7, GSTA1, GSTA2, GSTA3, GSTA4, GSTA5, GTF2A2, GTPBP4, GTSE1, HELLS, HES4, HES5, HGS, HINT2, HKDC1, HMSD, HOMER1, HRCT1, HYAL1, HYAL2, ICK, IDI1, IDI2, IL11RA, IL17A, IL17F, IL32, IMMT, INS-IGF2, IQCE, IQSEC2, IRS1, ISG15, JMY, KBTBD12, KCTD11, KCTD18, KDSR, KIAA1045, KIAA1468, KIAA1539, KIAA1751, KIF5C, KLHL17, KLHL30, LAMC3, LARP4B, LCAT, LCN10, LDHAL6B, LFNG, LHPP, LIPC, LOC441869, LOC643988, LPO, LRFN4, LRRC45, LYPD6, LYPD6B, MAD1L1, MAMSTR, MBD5, MCM2, MCM3, MED27, MELK, METTL10, MGLL, MIB2, MKS1, MMEL1, MMP23B, MORN1, MPO, MPP4, MRI1, MRPL12, MRPL20, MRPS23, MSH3, MSI2, MSMP, MTRNR2L2, MTX3, MXRA8, MYO1E, NADK, NAPRT1, NARFL, NDUFA4L2, NDUFB3, NEU4, NIF3L1, NKX1-2, NLGN3, NOC2L, NOG, NOS2, NPB, NPLOC4, NPR2, NPTX1, NTNG2, NUDT1, NUP214, OAT, ODF3L1, OPA3, OR13J1, OR2S2, OR4D1, OR4D2, ORC2, ORC4, P4HB, PABPN1L, PADI3, PANK4, PAPD4, PAQR8, PCYT2, PDE6G, PDIA2, PDYN, PEX10, PHGR1, PHKG1, PHLPP1, PIGN, PIGO, PKDREJ, PKHD1, PKN1, PLA2G1B, PLCH2, PLEKHN1, PLXNA1, PODXL2, POMT1, PPAPDC3, PPARA, PPIL3, PRAP1, PRDM12, PRKACA, PRKCZ, PRPH, PRRC2B, PRSS8, PTX4, PUSL1, PXDN, QRFP, RAB7A, RAPGEF1, RASGRF2, RECK, RER1, RGP1, RGS12, RNF111, RNF213, RNF223, RNF38, RNF43, RNPS1, RPL7L1, RPN1, RPTOR, RUSC1, RUSC2, RUVBL1, SAMD11, SCNN1D, SCPEP1, SDF4, SDK1, SEC61A1, SEMA6B, SEPT12, SERINC5, SERPINB10, SERPINB11, SERPINB12, SERPINB13, SERPINB2, SERPINB3, SERPINB4, SERPINB5, SERPINB7, SERPINB8, SETX, SGOL2, SGSH, SH3YL1, SHD, SHMT2, SIGMAR1, SIT1, SKI, SLC17A8, SLC25A10, SLC26A11, SLC35E2, SLC35E2B, SLC38A10, SLC38A5, SLC6A19, SLTM, SMAD6, SNTG2, SNX8, SOAT2, SOD3, SPAG8, SPATC1, SPATS2L, SPZ1, SRSF1, SSU72, STOML2, STRA13, STRADB, SUPT4H1, TAS1R3, TBC1D16, TBC1D22A, TCP10L, TELO2, TESK1, TFF2, THBS4, THOC4, TLN1, TLR3, TM2D1, TMEM105, TMEM132A, TMEM14A, TMEM18, TMEM216, TMEM237, TMEM52, TMEM88B, TMEM8B, TMEM95, TNFRSF11A, TNFRSF14, TNFRSF18, TNFRSF4, TPM2, TPM4, TPO, TPRA1, TRAK2, TRAM2, TRIM25, TRIM3, TRMU, TSHZ1, TSPAN10, TTC34, TTC38, TTF1,

TTLL10, TTYH3, TUBB8, TUSC2, UBE2J2, UCK1, UNC13B, UPK3B, VCP, VEZF1, VPS4B, VWA1, WBSCR17, WDR37, WDR90, WNT7B, ZADH2, ZCCHC2, ZFYVE16, ZMYND11, ZNF205, ZNF407, ZNF410, ZNF808, ZRANB1.

SUPPLEMENTARY REFERENCES

1. Goodarzi, H., Elemento, O. & Tavazoie, S. Revealing global regulatory perturbations across human cancers. *Mol. Cell* **36,** 900–11 (2009).

2. Ashburner, M. *et al.* Gene ontology: tool for the unification of biology. The Gene Ontology Consortium. *Nat. Genet.* **25,** 25–29 (2000).

3. Oakes, C. C. *et al.* Evolution of DNA methylation is linked to genetic aberrations in chronic lymphocytic leukemia. *Cancer Discov.* (2013). doi:10.1158/2159-8290.CD-13-0349
